# Scaling up resource recovery of plastics in the emergent circular economy to prevent plastic pollution: Assessment of risks to health and safety in the Global South

**DOI:** 10.1177/0734242X221105415

**Published:** 2022-07-23

**Authors:** Ed Cook, Costas A Velis, Joshua W Cottom

**Affiliations:** School of Civil Engineering, University of Leeds, Leeds, UK

**Keywords:** Plastic, solid waste, health and safety, Global South, resource recovery, circular economy

## Abstract

Over the coming decades, a large additional mass of plastic waste will become available for recycling, as efforts increase to reduce plastic pollution and facilitate a circular economy. New infrastructure will need to be developed, yet the processes and systems chosen should not result in adverse effects on human health and the environment. Here, we present a rapid review and critical semi-quantitative assessment of the potential risks posed by eight approaches to recovering value during the resource recovery phase from post-consumer plastic packaging waste collected and separated with the purported intention of recycling. The focus is on the Global South, where there are more chances that high risk processes could be run below standards of safe operation. Results indicate that under non-idealised operational conditions, mechanical reprocessing is the least impactful on the environment and therefore most appropriate for implementation in developing countries. Processes known as ‘chemical recycling’ are hard to assess due to lack of real-world process data. Given their lack of maturity and potential for risk to human health and the environment (handling of potentially hazardous substances under pressure and heat), it is unlikely they will make a useful addition to the circular economy in the Global South in the near future. Inevitably, increasing circular economy activity will require expansion towards targeting flexible, multi-material and multilayer products, for which mechanical recycling has well-established limitations. Our comparative risk overview indicates major barriers to changing resource recovery mode from the already dominant mechanical recycling mode towards other nascent or energetic recovery approaches.

## Graphical abstract


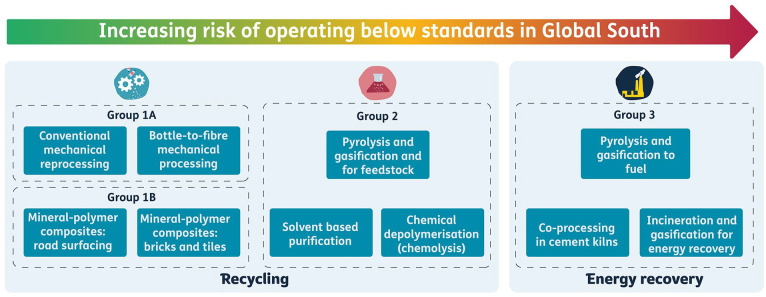


## Introduction

In recent years, several voluntary commitments have been made by fast-moving consumer goods (FMCG) companies in an effort to enable more post-consumer plastic packaging waste to be collected and recycled. For instance signatories to the Plastic Pact (Ellen MacArthur Foundation, 2017) have pledged to include post-consumer recycled plastic in plastic packaging they place on the market by 2025. It is anticipated that these initiatives will reduce the amount of plastic waste that is mismanaged by being dumped on land, into water or through burning in open uncontrolled fires, thereby preventing plastic pollution (Lau et al., 2020).

If these voluntary commitments to the circular economy are successful, a large mass of additional material will need to be processed and converted into new raw materials or energy worldwide (Ellen MacArthur Foundation and UN Environment Programme, 2020). Research conducted by Lau et al. (2020) proposed high-level solutions that focus on reduction, material substitution, collecting, processing or disposing of this large mass of material, but as yet there are no comprehensive efforts to assess their safety when implemented at global scale.

In the absence of consolidated evidence, as part of their voluntary commitments, FMCG companies, non-governmental organisations and sector specialists have been investigating existing resource recovery ‘approaches’ (opportunities) to recovering value (materials or energy) from plastic packaging (Stewart et al., 2018). Some of these approaches include those that recover the material whilst preserving its physical and chemical structure and integrity such as (Ellen MacArthur Foundation, 2021): A1) Conventional mechanical reprocessing for extrusion; A2) Bottle-to-fibre mechanical reprocessing for extrusion; A3) Mineral–polymer composites: road surfacing and brick and tile production and A4) Solvent-based purification (CreaCycle GmbH, 2015) (Table 1). Other approaches involve decomposition of chemical structures in plastics to create new chemicals that have the potential to be synthesised into starting materials for new plastics production such as A5) Chemical de-polymerisation (chemolysis) and A6) Pyrolysis or gasification (Hundertmark et al., 2018).

**Table 1.** Engineering approaches to recovering value (resource recovery) from post-consumer plastic packaging that has been collected for recycling.

Approach number	Approach name	Description
A1	Conventional mechanical reprocessing for extrusion	Plastic wastes are sorted, purified, comminuted and re-melted (extruded) into pellets for conversion (Shen et al., 2014)
A2	Bottle-to-fibre mechanical reprocessing for extrusion	Polyethylene terephthalate (PET) waste is sorted, purified, comminuted and re-melted (extruded) into polyester fibre for use in textile production (Shen et al., 2011)
A3 (a and b)	Mineral–polymer composites: road surfacing (A3a) and brick and tile production (A3b)	Plastics wastes are melted alongside minerals, such as sand or aggregate, acting as a binding and strengthening agent when cooled (Kumi-Larbi et al., 2018; Zhu et al., 2014)
A4	Solvent-based purification	Solvents are used to dissolve specific polymers in plastics, enabling them to be separated from additives and residues (Ügdüler et al., 2020)
A5	Chemical de-polymerisation (chemolysis)	Plastics are reacted under heat and pressure alongside catalysts to depolymerise them and enable the resultant monomers and oligomers to be used in primary plastics production (Raheem et al., 2019)
A6	Pyrolysis or gasification	Plastics are heated, without (pyrolysis) or with limited (gasification) oxygen, resulting in polymer chain scission and reformation of hydrocarbons as gases solid or liquid that can be used as fuels or as feedstock for repolymerisation (plastic production) (Lopez et al., 2017; Ragaert et al., 2017)
A7	Co-processing in cement kilns	Waste is used as a replacement fuel for coal in cement production plants (also often termed: waste-derived fuel, solid-recovered fuel – in which case it is often applied to material derived from mixed waste rather than that which has been collected for recycling) (Velis et al., 2012)
A8	Incineration with energy recovery	Waste is combusted whilst heat is recovered to heat space and water whilst electricity is generated (Neuwahl et al., 2019)

Other approaches explored by FMCG companies are typically referred to as ‘recovery’. These recovery approaches are not considered ‘recycling’ according to globally applied terminology (Supplemental Table S8), but are included here because, correctly or not, they are perceived to be desirable options in a cascading circular economy, these are: A7) Co-processing in cement kilns (Brock et al., 2021; Jiao, 2020; Republic Cement, 2020) and A8) Incineration (combustion) with energy recovery (often described as ‘energy-from-waste’ (EfW) – but hereafter ‘incineration’). A7 and A8 have been used to process material that has been collected from the mixed municipal waste stream, but due to the composition of the plastics and/or lack of available markets, they are unsuitable for mechanical reprocessing.

Many of these approaches are, or are planned to be, implemented in Global South where the majority of the world’s plastic packaging is mismanaged (Cook et al., 2020a). However, some countries lack well-resourced, independent environmental and safety regulators, and there are concerns that emissions from some processes may result in harm to human health and the environment (Nguyen et al., 2021; Rollinson et al., 2019; UNEP and Basel Convention, 2020).

Several efforts have been made to compare the relative safety of potential approaches to managing post-consumer plastic packaging that has been collected for recycling. For instance, a working group of parties to the Basel Convention on the Control of Transboundary Movement of Hazardous Wastes and Their Disposal (hereafter, the Basel Convention) has drafted guidance that begins to describe the safe treatment of plastic waste (UNEP and Basel Convention, 2020). The purpose was to support Decision BC-14/13 of the Convention that compels parties to ensure exported plastic wastes undergo ‘environmentally sound management’ in the country of destination.

Other comparisons of approaches to recovering value from post-consumer plastic packaging waste that has been collected for recycling focus on certain aspects of environmental harm or human safety. Lazarevic et al. (2010) reviewed studies that compared the life cycle impacts of managing plastic waste using mechanical reprocessing, feedstock recycling, incineration and landfill. The study did not assess the impact on human health, but found that mechanical reprocessing was the least impactful on the environment. More general, reviews exist such as Crippa et al. (2019) and Ragaert et al. (2017), who provide descriptions and process information that explain the potential pathways for processing plastic wastes. In each case, the focus is on the resource recovery phase of the circular economy; however, only summary evidence is provided that relates to the potential environmental and public health risks from the various processes.

There are many other sources that address the public, occupational and environmental safety of approaches to recovering value from plastic waste; however as yet, there is no review that compares the approaches or assesses their suitability for implementation or continued existence in the Global South. For clarification, the term ‘Global South’ is used here for convenience but alludes to countries where the lack of effective, independent, well-resourced enforcement and regulation might result in an elevated risk to human health and the environment.

Here, we rapidly review such evidence for eight types of resource recovery approach, each of which is presented in their own section (Table 1). Evidence is summarised in sub-sections for (1) Overview – prevalence and commercial maturity; (2) Risks to the environment, particularly global warming and (3) Risks to occupational and public health. Finally, we compared the approaches and qualitatively assign scores to indicate the comparative risk to human health and the environment when implemented in the Global South.

For clarification, this review is concerned with the eight engineering approaches to resource recovery as they are applied to treating post-consumer single-use plastic packaging that has been collected for recycling. This means that post-industrial (pre-consumer) material is excluded as well as mixed wastes that have not been collected for recycling. The approaches included are those that, via industrial partners, have been utilised by or considered for utilisation by FMCG companies. This means that some approaches that are more nascent, at bench scale and of very low technological readiness (U.S. Department of Energy, 2011), such as enzymatic conversion (Tournier et al., 2020) or hydrothermal carbonisation (Shen, 2020) had to be excluded. Other parts of the waste system are also excluded from this review, such as waste collection systems (formal and informal), reuse, minimisation and material substitution.

## Methods

### Literature review

Literature was obtained by searching peer-reviewed content in the archival database Scopus, Google Scholar (e.g. non-governmental organisation and industry led reports) and generic internet content via Google. A rapid search was carried out for existing reviews of each of the approaches (Table 1). However, drawing on evidence only from the reviews would have introduced bias to the study and an over-reliance on the robustness of a third party’s investigation. To establish a reliability pedigree, and inform inclusion/exclusion, samples of articles cited in each review were checked to ensure that the findings of original works had been correctly and fairly represented. If there was an indication that this was not the case, further samples were taken and if necessary the review was rejected for inclusion. Other considerations included the number of times a review had been cited by others in the context of the publication date, whether a report was funded by a particular narrow group of stakeholders and the quality and thoroughness of interpretation of data by the author of the review.

Citation/snowball search methods (Cooper et al., 2018) were used to identify more recent studies carried out since reviews had been conducted. In some cases, no relevant reviews exist, for instance for plastic packaging co-processed in cement kilns; therefore, relevant individual papers were assessed.

### Visual assessment of online media

As there is hardly any relevant process data (e.g. process system inputs, outputs, throughput, energy use, emissions, workforce behaviour and safe systems of work) for many of the topics covered in this review, an assessment of online multimedia (video) sources was carried out to identify risks to occupational health and safety from mechanical reprocessing and mineral–polymer composite slab and tile production. Observations of multimedia content have emerged in the last decade following the rising popularity of video sharing platforms such as YouTube (Kousha et al., 2012). As we were not able to find prior research that assessed occupational health and safety from multimedia evidence for the resource recovery topic, we developed our own novel rapid method by adapting a method from an observational study of safe systems of work employed by firefighters (Kahn et al., 2014) and one of recreational jumping from height into water (Moran, 2014).

Searches were carried out in the YouTube repository, which is the most widely used repository with the widest scope (Tackett et al., 2018) using a variety of terms including ‘plastic recycling’ and ‘plastic and sand tile production’. National terms were added to the search operators such as ‘India’, ‘China’ and ‘Brazil’. Other national search terms for countries that are highly industrialised with large amounts of municipal solid waste generation were also tested but yielded no useful results – this process was not systematic. The purpose of this part of the study was to obtain an indication of good or poor practice, working conditions, engineering controls to manage occupational and public risk and identify potential extremes of occupational safety behaviour. We did not attempt to determine the prevalence of these practices because the method feasible was not deemed as suitable. To control for bias, we chose to exclude footage intended to indicate bad practice as there was a risk that fill makers had cherry-picked poor behaviour. Instead, footage that was intended to demonstrate or ‘showcase’ a process or existing operation was assessed.

For each video, basic information was recorded (Table 4), and the main hazards were identified using Hughes et al. (2016) and consolidated into the following list:

1. Unguarded fast or high-torque machinery in close proximity to workers
2. Worker interaction with machinery resulting in risk of being drawn in
3. High-temperature equipment in close proximity to workers’ risk of burns
4. Risk of interaction with unknown potentially hazardous materials or substances (i.e. through atmosphere, dermal contact or ingestion)
5. Risk of burns from caustic substance
6. Particle loss to the environment likely
7. Risk of aerosolised hazardous substance
8. Risk of ballistic injury to hands, feet and body from interaction with sharp or heavy objects.

### Inclusion/exclusion criteria

Technical literature and other sources of information identified were assessed for inclusion in this study according to the criteria listed in Table 2.

**Table 2.** Inclusion and exclusion criteria.

Inclusion	Exclusion
Conventional plastics	Waste collection – for example, waste pickers
Technologies listed	Biodegradable plastics
Supply systems	International trade in plastic scrap
Post-consumer plastic waste	Post-industrial waste
Packaging	Non-packaging
Peer-reviewed journal articles, conference papers, books, reports, websites and online multimedia	Reuse/alternative delivery systems
	Film footage intended to expose poor practice

### Assessment criteria for approaches

#### Commercial and technological maturity

The commercial and technological maturity of each approach alongside its commercial prevalence provides valuable insights into the level of risk of safe operation. Although well-established processes benefit from many years or even decades of experience, there may be an increased risk of harm to human health and the environment from more nascent approaches due to hazards that were not expected or sufficiently mitigated. The technological readiness level (TRL) scale (U.S. Department of Energy, 2011) is commonly used to indicate how far a technology has progressed towards commercialisation. However, once an innovation has reached level 9, the scale does not indicate whether the technology is commercially sustainable in the real world, only that it is stable and functional at a large enough scale to be commercialised.

Though our objective differs from a proposal by Bruno et al. (2020) that extends the TRL scale to consider legal, organisational and societal readiness, we have used this as a basis to create our own four-level scale for low–high maturity (Supplemental Table S1).

#### Risk of harm to the environment and human health

The risk of harm to the environment and human health were described and summarised in Supplemental Table S6. As the majority of robust data exist only for the Global North context, these were assessed first. Scores for the Global South were then adapted from the Global North using objective reasoning to infer likely conditions, and controls to protect human health and the environment. The descriptions in Supplemental Table S6 were then ranked using criteria adapted from Velis et al. (2021) according to four levels: low risk, medium-low risk, medium-high risk and high risk, where the evidence was insufficient to make an assessment, no score was given.

#### Risk of operating below standards (appropriateness)

Each approach to managing plastic packaging waste in the resource recovery phase was scored for its appropriateness for safe operation in the Global South using the matrix in Table 3. The score was chosen at the lowest intersection of the two scores for either environmental or human health risks on the *x*-axis and the maturity on the *y*-axis.

**Table 3.** Matrix for assessing risk of operating below standards in the Global South (appropriateness).

		Risk to either environment or human health in the Global South (whichever is greatest)				
		L	ML	MH	H	ID
Commercial and technological maturity	H	L	ML	MH	H	ID
	MH	ML	ML	H	H	ID
	ML	ML	MH	H	H	H
	L	ML	MH	H	H	H
	ID	ML	MH	H	H	H

Risk of operating below environmental and health standards in the Global South (appropriateness): L: appropriate/low risk of operating below standards; ML: appropriate but with some risk of operating below standards; MH: inappropriate but could be implemented if operating standards sufficient; H: inappropriate/high risk of operating below standards; ID: insufficient data to make an assessment.

#### Grouping of approaches

Each of the eight approaches and sub-approaches were arranged into groups according to the characteristics: maturity, risks to human health and environment and of operating below environmental and health protection standards in the Global South (appropriateness). Three groups emerged (G1–G3), one of which was divided into G1a and G1b due to slightly differing levels of evidence and maturity. Approach 6 was divided into two sub-groups during this process to demarcate between pyrolysis and gasification used for feedstock and fuel; the former of which is immature and the latter of which is mature but with questionable environmental benefit when applied to post-consumer waste plastic packaging.

## Approach 1: Conventional mechanical reprocessing for extrusion

### Overview

In the Global South, mechanical reprocessing of plastics has been carried out since the 1980s (Lardinois et al., 1995; Wahab et al., 2007), long before many high-income countries developed commercial reprocessing capacity. Despite these several decades of activity, very little published data exist on how these processes are carried out in the Global South context, which makes it challenging to assess the risk to human health and the environment.

In the Global North, plastics reprocessor operations are reasonably well-documented, though commercial confidentiality may sometimes obscure the latest developments. In this setting, manual sorting is slowly being replaced as sensor-based separation technology increases in accuracy and many modern plants have reported to reduce their material losses substantially as their processes and learning mature.

Mechanical reprocessing of post-consumer waste plastics involves several steps aimed at purifying and standardising polymers and their additives to make them suitable for remelting and forming into new products (Schyns and Shaver, 2021). Reprocessors of waste packaging often start with a feedstock that is more-or-less a single polymer, though other materials and objects such as closures, labels, glues, inks and residues of materials and substances from the items’ use such as food or beverages may also be present. Materials and substances may also become adhered or attached to packaging during handling and sorting during the use phase. For instance, a plastic bottle that has been collected, mixed and compacted in a waste collection vehicle and deposited on a dumpsite may exhibit surface contamination with food and dust, or has materials such as paper or metal trapped within its folded structure (Gall et al., 2020).

‘Contaminants’ are removed during several sorting steps that prepare the material for reprocessing including size reduction (comminution), separation by density (sink/float), washing (either hot, cold and or with chemicals) and optical separation (near infra-red or laser) (Vogt et al., 2021). A manual sorting step is almost always included at some point between collection for recycling and reprocessing, because mechanical means are rarely sophisticated enough to obtain sufficient material quality to make new products. There are a multitude of configurations of waste plastic reprocessing operations from the highly sophisticated facilities common in Europe, some of which employ robotics and automated quality management systems, to extremely rudimentary operations seen in some areas of the Global South (Neo et al., 2021).

### Environment

#### Global warming potential

The majority of life cycle assessment (LCA) studies of post-consumer plastic waste management support the ranking system offered by the ‘Waste Hierarchy’ (European Commission, 2008). Lazarevic et al. (2010) reviewed 77 scenarios reported in 10 LCA studies that evidenced impacts of mechanical reprocessing, feedstock recycling, incineration and landfill to compare the relative burdens across six LCA impact categories. Compared to incineration, mechanical reprocessing showed a lower abiotic depletion potential, energy use and global warming potential findings that were supported more recently by Bernardo et al. (2016).

An exception highlighted by Lazarevic et al. (2010) was evidence from Frees (2002), indicating that high levels of surface contamination with ‘organic’ (hereafter ‘biological’) material resulted in substantial burdens associated with hot water washing, wastewater treatment and plastic waste pretreatment. According to Lazarevic et al. (2010), these burdens increased the emissions from mechanical reprocessing to more than those emitted by incineration. However, a contrary result was reported by Krogh et al. (2001) who reported that despite the high biological material contamination (7% – by weight – wt), mechanical reprocessing had 100% better overall performance compared to incineration. Theoretically, the addition of sodium hydroxide to the washing process should negate the need for hot water use; however, there is evidence that both heat and the caustic additive are used in combination as reported in previous LCA study (Aryan et al., 2019). Importantly however, very few LCAs investigate reprocessors operating in a Global South context, meaning that process data are absent for these facilities (Laurent et al., 2014a). A few exceptions (non-exhaustive search) exist for China (Gu et al., 2017; Zhang et al., 2020), India (Aryan et al., 2019; Choudhary et al., 2019) and Brazil (Martin et al., 2021).

#### Water use

Clearly water consumption is a concern in countries where it is scarce; however, according to Laurent et al. (2014b), it is reported in less than 15% of LCA waste management studies. As a guide to the magnitude of water consumption during mechanical reprocessing, Chen et al. (2019) provided water depletion potential factors for the mechanical reprocessing of several polymers, indicating between 340 and 452 L t^−1^ material processed and waste-water discharge of between about 65 and 95%, depending on the polymer being processed (Supplemental Table S2). Aryan et al. (2019) reported much higher water use: 1200 L t^−1^ for polyethylene (PE) milk pouches and 1600 L t^−1^ for PET reprocessed at a comparatively small–medium-scale facility in India. The water use reported at the plant not only contributed to freshwater aquatic ecotoxicity but also to marine aquatic ecotoxicity and eutrophication. Moreover, the plant used coal to heat the water and dry the pouches in the winter when the sun provided insufficient heat.

The rapid review of YouTube studies carried out here (Table 4) observed the use of sodium hydroxide in four sources, mainly those that were more technologically sophisticated, handling large quantities of post-consumer material. However, eight of the processes observed did not clean material using water; instead, mainly secondary plastic foils were fed either directly into extruders or via a comminution step. It is unclear how common this ‘dry processing’ is, but it has been reported to be carried out by Aryan et al. (2019) in India and has been observed at Biffa Polymers in the UK (Houston, 2019, personal communication).

**Table 4.** Summary of practices involving plastics reprocessing in the Global South observed on multimedia posts with identification of hazards and hazard mitigation practices.

Source	Context	Feedstock	Process description	Dry/wet	Soph. cat.	Hazards								Mitigation measures or comments
						1	2	3	4	5	6	7	8	
Daharwal (2018)	Nagpur, India	Plastic bags	Manual separation, comminution, particle manually moved to extruder and made into mini-football sized lumps of polymer	Dry	Low	x	x	x	x			x	x	• No obvious SSW • No PPE use • No guarding on equipment
Mooge Tech. (2015)	Assumed China	PET packaging	Co-located sorting and reprocessing system including de-baling, trommel, de-labelling (rotating star), electromagnetic separation, high-speed friction washing, centrifugal dewatering, comminution, float sink separation	Wet	Medium-high	x	x						x	• Only one worker observed wearing gloves to sort manually, possibly drawing in risk on several conveyors though film not detailed enough to assess this
Triwood1973 (2009)	China	PET bottles	Polyester spinning includes comminution, float sink separation, washing, drying and extrusion into fibres which are then carded and woven into polyester sheet	Wet	Medium-high	x				x	x		x	• PPE – face shield used • Gloves used by some workers • Possibly insufficient respiratory protective equipment used during carding
sps (2018a)	India	LDPE bags	Clean, dry bags fed by hand into extruder which produces strands that are cooled and cut into pellets	Dry	Low	x	x	x					x	• No SSW in place • Stick to feed material in to extruder prevents drawing in • Open top sandals around molten plastic
sps (2018b)	India	LDPE bags	Clean, dry bags sorted by hand and cleaned with a knife then fed by hand into extruder which produces strands that are cooled and cut into pellets			x	x	x			x		x	
SS IndustrieS (2019)	India	Hard plastics	Material manually loaded in baskets into shredder	Dry	Low	x	x					x	x	• No obvious SSW • No PPE used and all in open top sandals • No guarding on equipment • Women help each other with heavy loads
Saha (2020)	Bangladesh	Misc. coloured PET packaging	Hand sorting and grading of material on the floor with low sided baskets followed by comminution, washing and sink float separation where material skimmed by hand. Appears to be a hot wash but not obviously so	Wet	Medium	x	x	x			x		x	• No obvious SSW • No PPE used and all in open top sandals or bare feet • No guarding on equipment
The Times of India (2019)	India	Hard plastics	Material crushed and extruded with durability additives then pelletised and re-melted and formed into tiles	Dry	Low-medium	x	x	x					x	• No obvious use of PPE though few workers shown
Kao (2014)			Manual de-baling and summary sort before inclined conveyor feeds trommel. Process then includes more intensive manual sorting, a hot wash (possibly caustic) – film seemed to stop prematurely	Wet	Medium					x				• Stop rope above conveyor • Gloves worn by approximately 50% of staff • Some use of face coverings • Some use of hearing protection • Evidence of SSW
Carretino Proyectos (2016)	Sriracha Thailand	Plastic packaging	Mechanical loading of bales into automated de-baler followed by caustic wash and drying tumbler, lights separated with cyclone then missed labels removed manually (special tool) on conveyor. Comminution followed by sink float separation to remove closure materials followed by another caustic wash and drying tumbler, yet another sink float carried out before high purity achieved. Final manual polishing to increase purity, no extrusion here	Wet	Medium-high	x	x		x	x			x	• Huge sign on front of building states ‘safety first’ • Signage throughout building warning of danger • Gloves worn by all workers • Face coverings worn by ~20% of workforce, presumably according to need • High level of safety evident
Micro Machinery Manufacture (2018)	India	Woven PP raffia sacks	Hand sorted before being hand fed directly into extruder dry – extruded into strands and pelletised (possibly this is a display machine)	Dry	Medium	x	x	x						• No obvious SSW • No PPE use and all operatives in bare feet • Very little guarding on equipment
Kumar (2019)	South Asia (assumed)		Dry fed shredder created flake, which was manually fed into an extruder and pelletised	Dry	Low	x	x	x						• No obvious SSW • No PPE use • No guarding on equipment
Potdar (2015)	South Asia (assumed)	Electrical insulation cables	Apparently are course shredded and then manually transferred to a hoper and extruded into strands	Dry	Low-medium	x	x	x	x		x	x	x	• No obvious SSW • No PPE use • No guarding on equipment
Singh (2018)	India	Mixed plastic packaging	Comminuted and mixed in a dark environment where workers manually load between unit processes including washing, drying and extrusion	Wet		x	x	x	x	x	x	x	x	• No obvious SSW • No PPE use • No guarding on equipment • Very dark and terrifying conditions
GlobeTrotter (2013)	Ahmedabad, India	Pipes, polythene moulding materials	Material is handfed directly into extruder stranded and pelletised 1.8–1.9 tonnes d^−1^	Dry	Low-medium								x	• No obvious SSW • No PPE use • No guarding on equipment

Hazard codes as follows: (1) unguarded fast or high-torque machinery in close proximity to workers; (2) worker interaction with machinery resulting in risk of being drawn in; (3) high-temperature equipment in close proximity to workers risking burns; (4) risk of interaction with unknown potentially hazardous materials or substances; (5) risk of burns from caustic substance; (6) particle loss to the environment likely; (7) risk of aerosolised hazardous substance; (8) risk of ballistic injury to hands, feet and body from interaction with sharp or heavy objects.

WEEE: waste electrical and electronic equipment; ELV: end-of-life vehicle; SSW: safe system of work; PPE: personal protective equipment; PET: polyethylene terephthalate; LDPE: low-density polyethylene; PP: polypropylene; Soph. cat.: sophistication category. The term ‘wet’ refers to process in which plastics were subjected to washing and or sink-float separation; the term ‘dry’ refers to processes in which no water was used other than for cooling strands for palletisation.

#### Management of residues

A concern highlighted in recent years is the management of residues (solid rejects) by plastics reprocessors in countries where mismanagement of waste in general is also reported to be high (European Environment Agency, 2019). The inference is that non-targeted for recovery materials (low-value plastics or those that are problematic to sort and concentrate and contraries) may be mismanaged by dumping on land, discharge into waterways and coastal waters and open burning (Lau et al., 2020).

Although there are no academic studies that provide systematically gathered evidence on the prevalence of residue mismanagement by reprocessors, several documentaries and news articles have highlighted the phenomenon with film footage and still photographic evidence (60 Minutes Australia, 2019; BBC News, 2020; CBC News, 2019; Fruhnert, 2014; Sky News, 2018) as well as reports such as Velis (2014). The existence of the problem is also inferred by increasing regulation to curb transboundary movements of post-consumer plastic recyclate (often termed ‘scrap’), particularly from high-income countries to industrialised nations in the Global South. For instance, there are indications that part of the rationale for the Chinese Authority’s virtual ban (hereafter the ‘Chinese import ban’) on plastic imports in 2018 (Ministry of Ecology and Environment, 2017) was to reduce the risk of residue mismanagement (Liang et al., 2021).

Following the Chinese import ban, considerable amounts of material has been diverted to other countries, such as Turkey and several South and Southeast Asian countries including Malaysia, Thailand, Vietnam, Indonesia, Taiwan and India (Resource Futures-Nextek, 2018). This has resulted in planned or actual tightening of import restrictions, as their respective authorities fear mismanagement of residues that they are unable to enforce (APL, 2018; Bedi, 2019; CMA CGM (Japan), 2018; Cotecna, 2018; Das, 2018; Government of India: Ministry of Environment Forest and Climate Change, 2016; ISRI, 2018; Liang et al., 2021; Resource Recycling, 2019; Staub, 2018a, 2018b, 2019). Moreover, the mismanagement of residues from imported post-consumer plastic waste was the motivating rationale of the parties to the Basel Convention, who implemented significant restrictions on the export of plastics from high-income countries to the Global South from January 2021 (Secretariat of the Basel Convention, 2019).

In addition to the mismanagement of residues, Operation Clean Sweep (2020), Boucher et al. (2017) and Cole et al. (2016) have reported that plastics reprocessors are likely to be a proportionally small but significant source of microplastic pollution as a result of comminuted flakes and spilled pellets that are discharged into foul and surface water drainage systems during reprocessing. There is still very high uncertainty over the magnitude of plastic pellet loss from reprocessors. For instance, Cole et al. (2016) estimated pellet loss from the plastics industry as a whole at between 5.3 and 53 billion pellets (105–1054 t) per annum from the UK alone, based on 4.8 Mt being processed (reprocessor inputs). Lassen et al. (2015) provided a very rough estimate for plastics processors in Denmark, estimating that between 0.0005 and 0.01% of production may be lost as microplastics to the environment.

The rapid review of multimedia evidence presented in Table 4 indicates that pellet loss was uncontrolled in at least 5 out of the 15 facilities observed. This assertion is based on the assumption that the facilities observed did not have closed circuit wastewater treatment, and that there was observable evidence of material being spilled and discharged into drainage.

### Health

#### Emissions to air

Emissions from extrusion of the main conventional plastics used in packaging, such as PP, PE, PET, high-density PE and LDPE are not thought to result in harm to human health if carried out at controlled temperatures and using relatively pure feedstock. A study by Unwin et al. (2013) of atmospheric emissions at 10 UK plastics extrusion facilities (PP, PE, acrylonitrile butadiene styrene, PET and polyvinyl chloride (PVC)) found extremely low and often undetectable concentrations of carcinogens in all cases. All the sites and processes investigated by Unwin et al. (2013) incorporated a variety of engineering controls, including forced air ventilation local exhaust ventilation and mechanical dilution.

In the Global South, ventilation may not always be provided in plastics extrusion facilities. For example, a review by Cook et al. (2020b) found examples of facilities investigated in China where only passive ventilation was provided as a control measure. Other examples were identified in the multimedia evidence presented in Table 4, where workers were in close proximity to extruders without any observable mechanical ventilation or personal protective equipment. In several cases, there was evidence that end-of-life vehicle (ELV) parts and electrical goods including PVC insulation were being extruded without any form of ventilation or respiratory protective equipment. Speculatively, insufficient ventilation may be a widespread reality where resources are limited, and there is a lack of sufficiently resourced, independent health and safety regulation.

Although the most common polyolefins and PET do not usually result in harmful emissions if ventilation is sufficiently controlled, there is some evidence that if materials are not sourced or sorted carefully, then they may be inadvertently mixed with other plastics such as polystyrene and PVC, both of which result in harmful emissions when extruded (Cook et al., 2020b; He et al., 2015). Moreover, if the polyolefins and PET have originated from a source that involves some potentially hazardous substances being added, for example electrical casings, then these substances may volatilise when heated, exposing workers. For instance, an investigation by Tsai et al. (2009) next to PP and PE extrusion facilities in Taiwan, detected phthalates in air samples indicating that PVC had contaminated the feedstock. In two other studies, Tang et al. (2014, 2015) detected brominated flame retardants in environmental media near to plastic packaging recycling plants in China. Though the source was potentially confounded, the implication from these findings is that plastics from ELVs and electrical equipment plastics were being reprocessed into secondary feedstock, regardless of the potential carcinogenic and environmentally persistent substances within. Moreover, Tang et al. (2014) found high concentrations of brominated flame retardants in hair samples of young people who may work in plastics extrusion plants within the local area.

#### Accidents

Accidents in plastics reprocessing facilities are not specifically reported by the International Labour Organization (2019); they are instead aggregated across the waste industry, often including water and utilities. There are also no specific reports in the academic literature. The multimedia evidence presented in Table 4 was used as a reference and highlighted some deeply concerning practices at small- and medium-scale reprocessors operating in the Global South, particularly in India and China. For instance, evidence of the potential for workers to become entangled or drawn into fast-moving or high-torque machinery was observed at almost all facilities. In nine of the facilities observed, workers also carried out duties in very close, unprotected proximity to extremely hot machinery used for extrusion. The lack of robust and systematically obtained data on this topic makes it challenging to accurately assess, but clearly there are also some well managed plants in the Global South, where workers’ safety is systematically managed and emissions are controlled to protect public health; and this level of safety was apparent from the multimedia footage in 2 of 15 examples (Carretino Proyectos, 2016; Kao, 2014).

#### Food contact applications and legacy substances

Some plastics contain potentially hazardous substances that have been added intentionally or which have been unintentionally incorporated through adsorption, absorption, during production or conversion (Cook et al., 2020b). These substances are not usually bonded to the polymers themselves, but exist between the polymer chains as part of a mixture (Hahladakis et al., 2018; Wiesinger et al., 2021). These may migrate into the outside world from where they can disperse, or be absorbed into the body through the skin, via ingestion of food or through mucous membranes under certain conditions (Koch et al., 2009).

In much of the Global North and South, the use of hazardous substances in food contact packaging is tightly controlled by legislative frameworks designed to limit exposure to human health and the environment depending on the application (Cook et al., 2020b), for instance in India (Ministry of Health and Family Welfare, 2018). Thus, the use of recycled (secondary) plastics in food contact materials presents additional challenges because of the potential uncertainty around the origin of the material (primary production and manufacturing phase), its previous use (use-phase) and the way that it may have been handled, stored and treated after it has been used (end-of-life phase).

For example, to reduce the risk of fire, plastics used in many electronic goods are treated with flame retardants, which are potentially harmful to human health if absorbed into the body. If these electronic casings were used to make food packaging, there is a risk that they might migrate from within the polymer and leach into the food being packaged, potentially exposing the consumer. These substances are often described collectively as ‘legacy substances’ (Wagner and Schlummer, 2020). A summary of potential materials and substances that may exist in a secondary plastic is provided in Table 5.

**Table 5.** Summary of constituents in plastics; after Goulas et al. (2000), Hahladakis et al. (2018), Cook et al. (2020b) and Wiesinger et al. (2021).

Life cycle phase	Residual substance or substance group	Examples
Production and manufacturing	Polymers	• Polyethylene, PVC, PET
	Production residues	• Residual monomers (bisphenol A, styrene), dimers and oligomers • Residual catalysts
	Additives	• Plasticisers (e.g. di-(2-ethylhexylexyl)), fillers, brominated flame retardants (e.g. polybrominated diphenyl ethers)
Use	Food	• Cooking oil
	Household chemicals	• Pesticides • Paint stripper
End-of-life	Commercially used substances	• Engine oil • Battery acid

Legacy substances may occur in all secondary plastics; however, the concentration is usually so small as to be unlikely to pose any threat to human health (Wagner and Schlummer, 2020). Nonetheless, the potential risks are managed through stringent legislation in several countries. For instance, in India, the Ministry of Health and Family Welfare (2018) has recently prohibited the use of recycled content in food packaging under the Food Safety and Standards (Packaging) Regulations, 2018. Thailand, Japan and China have also historically implemented similar bans; however, there are indications that these laws may be relaxed to encourage more circular materials use (PackagingLaw.com, 2020; Rosato, 2020).

In Europe, the use of recycled content was banned in food contact packaging until fairly recently. However, Regulation EC 282/2008 (European Union, 2008) now allows secondary material use as long as certain conditions are met including requirements to:

- Use recycled plastic behind a ‘functional barrier’ as defined by Directive 2002/72/EC.
- Sort plastic to 100% efficiency, though where provenance is more certain, for example, from a kerbside separate collection (either comingled ‘single stream or separated ‘multi-stream’ collection), this requirement can be lowered on a case by case basis.
- Characterise input (feedstock) to determine if substances from misuse of the product are present (e.g. an orange juice container used to contain domestic pesticide).
- Obtain authorisation (from the relevant ministry) to use recycled content in conversion feedstock.

It is beyond the scope of this review to carry out a comprehensive review of regulatory frameworks managing the use of recycled content in food contact materials, but it appears that other countries allow its use including Mexico (PetStar, 2018), South Africa (Petco, n.d.) and Brazil (PackagingLaw.com, 2019).

## Approach 2: Bottle-to-fibre reprocessing

### Overview

Approximately 52 wt% of all textile fibres are polyester, representing 55 Mt of material produced in 2018 (Textile Exchange, 2019). Of this, 13% (7.2 Mt) was produced using a mixture of post-consumer PET bottles and post-industrial spun polyester fibre. Although the proportional and absolute mass of polyester produced from recycled content has increased steadily over the last decade, a reduction of 3% points took place following the Chinese import ban (Ministry of Ecology and Environment, 2017), highlighting the impact of international restrictions on the circular economy.

The use of secondary PET in polyester production has increased, alongside the amount of PET collected for recycling. But a greater proportion of the PET that is collected for recycling is now used in packaging (66%), whereas the remainder is used in bottle-to-fibre reprocessing (44%) (Park et al., 2014; Sarioğlu and Kaynak, 2018).

Polyester spinning does not differ greatly from other mechanical reprocessing systems for extrusion or blow moulding. It produces textile fibres that are as strong or in some cases stronger than its virgin counterparts as chain scission is substantially reduced in comparison to conventional mechanical reprocessing (Muslim et al., 2016; Shen et al., 2010).

### Environment

#### Global warming

Virgin polyester results in approximately 2.2–2.7 t CO_2_eq t^−1^ product (carbon dioxide, CO_2_) (Bartl, 2020) compared to, for example, cotton produced in China, which has been reported to be between 5.2 and 57.9 t CO_2_eq t^−1^ across the whole life cycle (Wang et al., 2015). However, LCA studies that evaluate the benefits of recycling PET into polyester fibre are limited, with just three relevant studies that have directly compared impacts, all of which showed a reduced emissions burden in comparison to virgin production (Komly et al., 2012; RDC-Environment, 2010; Shen et al., 2011).

In comparison with mechanical reprocessing for extrusion (Approach 1), bottle-to-fibre recycling has been found to have similar (Shen et al., 2011) or lower impact on global warming (Komly et al., 2012; RDC-Environment, 2010), mainly because the material that it replaces (virgin polyester and cotton) has very high burdens. These indicative results challenge the conception that bottle-to-fibre recycling, so-called open-loop, is less beneficial than bottle-to-bottle recycling, so-called closed loop because the output of bottle-to-fibre recycling (textiles) is not suitable for recycling in future (Geyer et al., 2016).

#### Water use

There are few clear estimates for water use from polyester spinning processes. Bartl (2020) suggested that primary polyester spinning uses 48.8 m^3^ t^−1^ water (excluding printing and dyeing) and Zhang et al. (2018) estimated 24.2 m^3^ t^−1^. It seems reasonable to assume that similar quantities are used for recycled PET, and cleaning and separation processes are similar to those carried out for conventional recycling. Possibly a more important comparator is for cotton, which has been reported to use between 2000 and 27,000 m^3^ t^−1^ produced (Bartl, 2020). Otherwise, there is no reason to assume that bottle-to-fibre reprocessing has a different water consumption rate in comparison to bottle-to-bottle reprocessing.

No specific data were found to indicate microplastic release from bottle-to-fibre reprocessing; however, it is reasonable to assume that it is the same as for other conventional recycling processes.

### Health

No evidence that has not already been discussed in Approach 1 was found to indicate specific health hazards from polyester spinning. Nonetheless, objective reasoning suggests that the use of only one polymer (PET) in bottle-to-fibre reprocessing, which is mainly used in packaging, may lower the risk of contamination from materials that have been used in other applications – for instance ELVs or electrical and electronic equipment.

## Approach 3: Mineral–polymer composites

### Overview

#### Road-surfacing (Approach 3a)

The use of waste plastics in road surfacing has been investigated as a solution to the plastic pollution crisis and could be used to recover unrealised value from the waste system (Chin and Damen, 2019; Wu and Montalvo, 2021). This potential has been embraced by some states in India (Karelia, 2018; Louise, 2019; News18, 2019), and the National Rural Roads Development Agency (n.d.) has developed guidelines for the use of plastic waste in roads.

To clarify, the roads discussed here are not made purely from plastic. Instead, the bitumen component is typically modified with around 5 wt% (2–10 wt%) plastic (Rødland, 2019). This means that when aggregate and sub-layers are included, the total mass of plastic used as a proportion of road construction mass is very small. Polymer modification of bitumen is well-established, having been investigated since the 1950s and has been in common use since the 1980s (Zhu et al., 2014), where it is used to improve elasticity reduced rutting, fatigue resistance, reduced thermal cracking and increased elasticity (Ahmadinia et al., 2011; Costa et al., 2013; Dalhat et al., 2017; Fang et al., 2014; Movilla-Quesada et al., 2019; RAHA Bitumen Co., n.d.; White, 2019; White and Reid, 2018). Until recently, it has been exclusively carried out using virgin polymers, including PE, PP, ethylene–vinyl acetate, ethylene–butyl acrylate, styrene–butadiene–styrene, styrene–isoprene–styrene and styrene–ethylene/butylene–styrene.

#### Bricks and tiles (Approach 3b)

The use of waste plastics as a bonding agent for the manufacture of tiles and bricks is becoming increasingly widespread, having been implemented by several charities operating in the Global South including WasteAid UK (Lenkiewicz and Webster, 2017). Several proprietary and open-source processes are available (Earth Titan, 2019) that involve melting plastic together with sand to form a paste, which is then pressed into moulds and left to cool. At its most basic, the process is carried out over a fire, whereas some processes observed were more automated (Kolev, 2019), including mechanical pressurised moulding, mechanical mixing and comminution of plastics with low-speed high-torque cutting mills (Earth Titan, 2019). In one example, sand was kiln-dried to improve the properties of the final product in advance of the plastic waste being added (NTVUganda, 2013).

Historically, there has been a paucity of published academic literature on brick and tile production using waste plastics, though several recent papers have explored the approach, finding it results in products with very high durability and strength (Ali et al., 2020; Salvi et al., 2021; Thorneycroft et al., 2018; Uvarajan et al., 2021). According to Kumi-Larbi et al. (2018) who tested the physical properties of LDPE-bonded sand, the compressive strength of the composite is greater than Portland cement sandcrete and similar to C20/25 concrete.

#### Unbound aggregate

Gu et al. (2016) reviewed 83 studies that investigated the use of plastics in concrete as a lightweight replacement for aggregate. Although it was not within the scope of the present review to assess this end-use, it is referred to here to identify it as a potential avenue of further research.

### Environment

#### Global warming

According to Wu and Montalvo (2021), very few studies have investigated global warming emissions from polymer modified asphalt, referring to just five studies that indicate mixed results from using waste plastics in comparison to virgin plastics. Four of these, Santos et al. (2018), Vila-Cortavitarte et al. (2018), Mukherjee (2016) and Nascimento et al. (2020), investigated plastics that are not commonly used in FMCG packaging (rubber, polystyrene, etc.). Only one, Poulikakos et al. (2017), investigated a range of waste plastics used in packaging, reporting substantial cost savings alongside a reduction in CO_2_ emissions. The model was highly theoretical and intended to demonstrate the potential concept rather than being based on physical implementation. It assumed that the plastics would not require substantial processing (cleaning and purifying) to be suitable for use in roads, reducing the environmental burdens associated with mechanical reprocessing, which was one of the comparators. Whether this uncertainty in material composition would be acceptable to road manufacturers is unclear.

There is insufficient data available to make an assessment over whether the use of waste plastics as a bitumen modifier provides overall environmental improvement across the life cycle. Intuitively, anything that reduces the need to resurface or replace roads using a product that would otherwise be wasted ought to provide some benefit. Given that there is substantial evidence to indicate that polymer modification of bitumen results in increased durability, it seems likely that its use would result in reduced maintenance and associated avoided burdens. Further investigation using post-consumer waste plastic packaging would provide more insight.

No LCA data were found for mineral–polymer composites used in the production of bricks, tiles or paving slabs. As this technology begins to increase in prevalence, it will be important to understand the full life cycle impacts. Clearly, rudimentary processing advocated by WasteAid uses very few resources (Lenkiewicz and Webster, 2017). The removal of plastic film would benefit the local environment though the process requires relatively clean sand, which would need to be sourced sensitively and sustainably (Torres et al., 2021). The LCA case is likely to be strongly driven by the avoided concrete production, which is an intensive sector with high energy demand (discussed further in Approach 7), but it is noteworthy that the heat used to melt the mineral–polymer mixture may be provided by open, uncontrolled fires. Therefore, the climate change impact of black carbon production may also have a significant effect on the overall environmental emissions (Reyna-Bensusan et al., 2018).

Fundamentally, both Approaches 3a (road surfacing) and 3b (brick and tile making) preclude further recycling of the plastics contained within, effectively consigning them to a single material cascade. This finality is classed by LCA protagonists as ‘open-loop recycling’ or widely elsewhere as ‘downcycling’ (Borrello et al., 2020; Tanguay et al., 2021). Both terms enable navigation of material circularity by a wide, often non-specialist audience. However, there is strong evidence that decision-making using hierarchical attribution of one process or another to an over-simplistic cascade level does not always result in the best overall environmental outcome (Geyer et al., 2016; Olsson et al., 2018). As discussed, the benefit to the environment of Approach 3 is related to: displacement of concrete or asphalt production; reduction of plastic debris at risk of emission to the terrestrial and marine environments and reduction in maintenance due to increased durability.

#### Particle emissions (microplastics)

One concern highlighted by Rødland (2019) is the potential for microplastic release from the polymer modified road surface during the use phase. The study reported microplastic emissions from each source in Norway at approximately 28 t y^−1^ polymer-modified asphalt, 90–320 t y^−1^ road marking polymer and 4250–5000 t y^−1^ from tyres. The source of most of the data reported by Rødland (2019) is Vogelsang et al. (2020) who acknowledged that there is huge uncertainty associated with the emission factors for polymer-modified bitumen, but the main particle emission source is likely to be studded tyres, used to drive through ice and snow in northern Europe, which abrade the road surface.

A potential risk is that the surface may become less durable if asphalt–polymer mixtures are incorrectly formulated, for instance too rich in polymer. It is recommended that this theory is investigated as lack of durability could influence both life cycle emissions and the risk of plastic particle emissions.

Intuitively, there may also be risks associated with microplastic release, fire risk and possibly migration of substances into the air inside buildings. No evidence was found for any of these, but it is suggested that this is considered in future investigations.

### Health

Asphalt is laid at 100°C–195°C (Nicholls et al., 2013). The top of this range of temperatures overlaps with the lower end of the range of temperatures used in mechanical reprocessing (Hahladakis et al., 2018). Given the evidence for potentially hazardous emissions from extrusion, it is conceivable that substances emitted during asphalt laying or brick and tile production could pose a threat to the health and safety of those who inhale air nearby, for instance if PVC, polystyrene or plastics from electrical goods or vehicles are used. There are no obvious concerns from the main conventionally used polyolefins and PET used in packaging and studies have found very low emissions from LDPE (Yamashita et al., 2009) and PE (He et al., 2015; Tsai et al., 2009).

Both White (2019) and White and Reid (2018), manufacturers of a proprietary modifier using discarded plastics (wastage) from recyclers in the UK, investigated air and leachate emissions from polymer-modified bitumen. Their air sampling found no hazardous emissions other than those that would be expected from the bitumen and no hazardous leachate was detected.

Discussions with Kumi-Larbi Jr (2020, personal communication) who has observed tile manufacture in West Africa revealed that the process often resulted in the plastics combusting briefly, which could potentially result in the production of substances of partial combustion. When completely decomposed under combustion, LDPE emits only water and CO_2_. However, the LDPE is likely to include small amounts of antioxidant and ultraviolet resistant additives and is unlikely to achieve complete combustion under very low heat used in the production process. Several relevant sources, including Valavanidis et al. (2008) and Barabad et al. (2018), reported low levels of particulate matter being emitted during LDPE combustion at low temperatures. The only other source of relevant information is from Wang et al. (2004) who reported a range of emission data from PE combustion.

#### Accidents

The multimedia evidence in Supplemental Table S3, highlighted several hazards associated with brick and tile production, including becoming entrained in high-speed or high-torque machinery and having contact with hot materials as they are formed and moulded to the shape of the tile or road surface.

## Approach 4: Solvent-based purification

### Overview

So-called chemical recycling technologies have received increasing attention from researchers who want to overcome the challenges associated with sorting and reprocessing the complex mixtures of polymers and additives found in post-consumer waste plastics (Davidson et al., 2021). These approaches have also generated huge interest from FMCG companies (Lee et al., 2021) who are interested to find new opportunities to recycle wastes that are unsuitable for mechanical reprocessing.

One technology is ‘solvent-based purification’, an approach that uses solvents to dissolve polymeric materials, allowing them to be separated from the additives and contaminants found in the plastics. There are seven groups of process according to Ügdüler et al. (2020): (1) shake-flask extraction, (2) Soxhlet extraction/batch multistage extraction; (3) ultrasonic extraction, (4) microwave assisted extraction, (5) supercritical fluid extraction, (6) accelerated solvent extraction and (7) dissolution-precipitation.

Mechanical reprocessing involves heating polymers, adding a thermal event to their history and causing some of the chains to break (chain scission/decomposition) and thus weaken the overall structure. Solvent-based purification keeps the polymer chains intact, thus creating a higher quality end product. However, the removal of solvents post-separation remains an issue in some processes, potentially hindering commercial viability (Zhao et al., 2018). Some authors argue that solvent-based purification should not be classified as ‘chemical recycling’ because the polymers are not completely deconstructed, and it should instead be classed as mechanical reprocessing (Crippa et al., 2019). As the solvents target specific polymers, the process should be applicable to the recovery of polymers from layered multi-material packaging (Kaiser et al., 2018; Walker et al., 2020) or even the plastic fractions in mixed material textiles such as polyester–cotton mixtures (Sherwood, 2020; Thiounn et al., 2020).

There is evidence of at least one pilot plant operating the CreaSolv^®^ and Newcycling^®^ process in Indonesia (Unilever, n.d.). The facility is reported to be capable of processing 3 t d^−1^ of water sachet waste per day (1000 t y^−1^) (Unilever, n.d.) and has aspirations to increase this to 30,000 t y^−1^. However, some recent evidence indicates that the plant failed to achieve viability and that it was closed in 2019 (Aliño et al., 2022). According to Crippa et al. (2019), no other commercially viable solvent-based purification facilities are currently operational. Ügdüler et al. (2020) agreed with this assertion, indicating that most processes are between TRL 3 and 8.

### Environment

As explained by Crippa et al. (2019), there is very little real-world process data available for solvent-based purification as the technology group is not yet commercialised. Ügdüler et al. (2020) carried out a basic LCA of two processes to remove additives; however, the work was highly theoretical, and it would be misleading to extrapolate further.

### Health

Though this technology does not exist commercially, the use of solvents, their treatment and disposal after-use is likely to be one of the most significant potential health concerns when this technology becomes commercialised. As discussed by Ügdüler et al. (2020), there are a multitude of solvents all of which are targeted at different additives. Many of these are potentially hazardous to human health such as chloroform, xylene, *n*-hexane and cyclohexane.

## Approach 5: Chemical depolymerisation (chemolysis)

### Overview

Approximately seven processes are grouped under the ‘depolymerisation’ category: (1) methanolysis, (2) glycolysis, (3) hydrolysis, (4) ammonolysis, (5) aminolysis, (6) hydrogenation and (7) alcoholysis (Kumar et al., 2011; Ragaert et al., 2017; Raheem et al., 2019). Plastics are reacted under heat and pressure with a range of substances including catalysts, acids, alkalis and alcohols causing the depolymerisation of the polymers (Raheem et al., 2019).

The most studied polymer for depolymerisation is PET (Crippa et al., 2019), which can be completely decomposed into its starting materials such as ethylene glycol, terephthalic acid bis(2-hydroxyethyl terephthalate) (BHET), or partially into dimers, and oligomers of the aforementioned. Glycolysis of PET is a commercially proven practice having been carried out for several decades by large chemical producers (Ragaert et al., 2017). Glycolysis of PET is suited to high-quality, post-industrial feedstock as shortcomings in the processes’ ability to remove dyes, copolymers and colourants make it unusable in other contexts.

Presently, glycolysis is only used to process off-specification post-industrial textiles and carpet fibres (Aquafil, 2014). The hope is that one day glycolysis can be used for processing post-consumer bottles, but as yet there is no evidence of this taking place at commercial scale (Crippa et al., 2019).

Several niche plastics can also be viably depolymerised with chemolysis such as poly(γ-butyrolactone) and aromatic polycarbonates; however, as Sardon et al. (2018: 381) explained, ‘plastics that can be so easily depolymerised lack suitable mechanical and thermal properties to be widely useful’.

### Environment

Given that there are only a handful of implemented facilities worldwide, it is unsurprising that there are few LCA studies that compare the environmental impacts of PET glycolysis with other technologies; only two are reported here. The first study by Shen et al. (2010) used data from Far Eastern New Century Co., a textile manufacturer who implements glycolysis of polyester fibres to BHET oligomers that are then re-polymerised to produce new PET. The process was compared with ‘semi-mechanical reprocessing’ (process data from the Long John Group) that involves sorting, flaking and palletising before re-extrusion, and full mechanical reprocessing where flakes are directly extruded into filament. The glycolysis resulted in higher costs and global warming emissions in comparison to the other two options, but still resulted in approximately half the global warming potential compared with virgin production. The study is based on highly specific industry data, which were incorporated at face value.

In a more recent LCA modelling effort by Meys et al. (2020), glycolysis of PET (described as ‘chemical upcycling’) is reported to perform slightly better than mechanical reprocessing, resulting in a comparative improvement of 1.13 t CO_2_eq t^−1^ processed.

Given the paucity of robust data and that the only two relevant studies are contradictory, there is no clear indication of the relative benefits of this process; it does not appear to have been used to process packaging anyway.

### Health

Assessing potential health implications of PET glycolysis is difficult in the absence of relevant data. As with any chemical processing, consideration should be given to controlling process emissions to protect the health of workers and the wider population.

## Approach 6: Pyrolysis or gasification

### Overview

#### Pyrolysis

Pyrolysis is a process that has been manipulated by humans for centuries to make charcoal from wood. Material is heated in the absence of oxygen, thereby preventing complete combustion. When applied to waste plastics, this results in a random scission and reforming of the polymer chains into a mixture of hydrocarbons resembling many of those found in crude oil in liquid (80 wt%) and solid (20 wt%) phase (Lopez et al., 2017; Ragaert et al., 2017). At scale, this is carried out at between 200°C and 1100°C (often around 500°C) and under moderate pressure (1–2 atm) (Mayer et al., 2019). The liquid fraction is often distilled into three basic fractions: kerosene, diesel and light oils (naphtha), and the solid material, known as char, includes non-combustible minerals and metals, as well as a high proportion of black carbon (Butler et al., 2011). More volatile substances are often flared as a firm of disposal or combusted to recover energy that is used to contribute to the heat necessary for the process.

The liquids from pyrolysis plants are all combustible, and according to Crippa et al. (2019), the most viable end-use for these is as fuel for ships and power plants. If sufficiently refined, pyrolysis oils can be used in higher-grade applications, such as road vehicles or aviation (Lopez et al., 2017). However, the ambition of many pyrolysis developers is to refine these oils into monomers and other compounds that can be used in primary plastic production (Papari et al., 2021).

The synthesis of plastic production feedstock using pyrolysis has the potential to both reduce the need to extract further fossil fuels and also to reduce the disposal and recovery burden on other parts of the waste management system (Hann et al., 2020). Moreover, if the process was able to compete commercially with mechanical reprocessing, the value of waste plastics would increase, creating a disincentive to mismanage plastics. Though pyrolysis innovation has accelerated in recent years, there is little evidence that pyrolysis oils have been used in the best-case scenario, which is to produce monomer feedstock (Solis et al., 2020). It is therefore assumed that the outputs of pyrolysis plants are being used as fuel.

Solis et al. (2020) reported that several plastic waste pyrolysis plants exist, and this indicates that ‘conventional pyrolysis’ is currently at level 9 of technological readiness based on the opinion of an academic expert. However, they also point out that there are few full-scale projects from which to determine economic feasibility. This leaves some doubt about how close these projects are to commercialisation. Khoo (2019) indicated that several plants exist including one in Japan (processing 15,000 t y^−1^) and two in the US of which one processes 25,000 t y^−1^ and the other is expected to process 100,000 t y^−1^ once operational. At time of writing, none of the plants reported by Khoo (2019) are verified as providing commercially proven processes.

Although it is possible that these plants can maintain a pyrolytic process, some serious doubts have been raised by Rollinson et al. (2019) and Rollinson et al. (2021) over whether any of these processes are self-sustaining or whether they require a constant external source of heat. Commercial sensitivities and lack of transparent process data may prevent these questions being answered, at least in the short term.

#### Gasification

Similarly to pyrolysis, gasification reactions involve the restriction of oxygen to allow decomposition of the polymers without complete combustion. Unlike pyrolysis, gasification takes place at higher temperatures (700–1200°C), and some oxygen is introduced into the process (Solis et al., 2020), resulting in partial oxidation of some hydrocarbons and atoms. Carbon monoxide (CO), hydrogen (H_2_), CO_2_, methane (CH_4_) and nitrogen (N_2_) are produced alongside some of the lower molecular weight hydrocarbons, such as ethane (C_2_H_6_) and ethylene (C_2_H_4_) (Ciuffi et al., 2020; Punkkinen et al., 2017). Collectively these are known as ‘syngas’.

Much heavier hydrocarbons are also produced resulting in a substance known as char alongside tarry substances. The tar is made up of a mixture of heterocyclic hydrocarbons such as pyridine and phenol, light aromatics such as benzene and toluene, polycyclic aromatic hydrocarbons such as naphthalene and heavier hydrocarbons that are not often characterised (Wolfesberger et al., 2009). This complex blend of substances is highly undesirable in the process as it quickly condenses, clogging and corroding pipework (Zeng et al., 2020). The char itself becomes contaminated by the tars, meaning it is unviable to clean, refine and utilise further (Benedetti et al., 2017; Lopez et al., 2018). The presence of these solids and their disposal continues to hinder the business case for gasification. Gasification of plastics produces less char compared to gasification of biomass or fibre (Sharuddin et al., 2016). However, the syngas itself tends to contain higher concentrations of char particulates; a key disadvantage to overcome when plastics are used as a feedstock (Lopez et al., 2018; Solis et al., 2020).

Historically, commercial gasification has used coal as a feedstock (Higman, 2013). Gasification plants that use waste as a feedstock have also begun to emerge over the last 10 years, but there is no comprehensive state of the industry review. Seo et al. (2018) reported four plants using waste as a feedstock in operation: one in Japan, one in Canada and two in Europe. Jayarama Reddy (2016) also reported multiple plants that were operational at some stage in the last 20 years, although it is clear that some have clearly ceased operating. In a more recent study, Solis et al. (2020) reported three gasification plants using plastics waste as a feedstock.

Hydrogen can be extracted from the syngas mixture, which can also be used to synthesise a range of substances including methanol and ammonia (Antonetti et al., 2017). Quicker (2019) indicated that gasification of homogenous mixed plastics had been shown to be viable at a German run plant. However, he cautioned that the plant has suffered from technical difficulties over many years and questioned the overall viability of the process. Even as a fuel production process, waste gasification becomes less viable due to the need to remove moisture from the syngas before it is combusted. Although synthesis of chemicals is the objective of gasification plants using coal as a feedstock (Ciuffi et al., 2020), Rollinson et al. (2020) suggested that this is unlikely to have happened at plants using waste as a feedstock. It is suggested that syngas from gasification is at best, converted into fuels; however, it is more likely that they are combusted directly in the plant, thus operating as an incinerator.

### Environment

The majority of studies that compare environmental performance of gasification and pyrolysis with other approaches to waste management do so on the basis of a mixed waste feedstock. Relatively few focus on plastics specifically. One exception is Khoo (2019), who compared emissions from mechanical reprocessing, pyrolysis, gasification and incineration in Singapore. Including avoided burdens, the life cycle emissions from pyrolysis were approximately 0.6–0.8 t CO_2_eq t^−1^ plastic processed in three studies and for gasification they were 0.4–0.9 t CO_2_eq t^−1^ plastic processed. Although both pyrolysis and gasification performed much better than incineration with energy recovery, they both resulted in higher emissions than mechanical reprocessing, which showed between 0.2 and 0.4 t CO_2_eq t^−1^ in three studies.

A most recent LCA presented by Schwarz et al. (2021) provided detailed life cycle carbon emissions data for 25 polymers processed using the methods: gasification and pyrolysis with and without monomer recovery, open and closed loop mechanical reprocessing, depolymerisation (for two polymers), dissolution (for 24 polymers) and incineration with and without energy recovery. Superficially, the study agrees with the differences between emissions from each technology reported by Khoo (2019), though the emissions were generally higher for pyrolysis, gasification and mechanical reprocessing and lower for incineration. Wherever pyrolysis and gasification were used for monomer production, emissions were generally slightly lower than those from mechanical reprocessing in the Schwarz et al. (2021) model. However, as stated this is entirely theoretical and there are no commercial plants available with which to validate such a model.

The study by Schwarz et al. (2021) was not carried out according to ISO14040, and the authors excluded sorting and collection emissions and modelled pure polymers (no additives) for simplicity. The exception was for two ‘case studies’ of (1) mixed PP and LDPE foils and (2) acrylonitrile butadiene styrene containing brominated flame retardants. These were used to ‘test’ and compare the counterfactual pure polymer results. Focusing on the mixed LDPE/PP foils, the case-study method assumed that material sent to pyrolysis would require as much sorting as that sent for mechanical reprocessing, and that material sent for gasification would require slightly less sorting. Residues were incinerated with energy recovery and assumed as 50 wt% for gasification and 59 wt% for mechanical reprocessing and pyrolysis. The outcome of this case study was that all the technologies more or less equalised in their life cycle emissions to a range between 3.2 t CO_2_eq t^−1^ plastic processed (gasification for monomer production) and 5.2 t CO_2_eq t^−1^ plastic processed by incineration with energy recovery.

The case studies reported by Schwarz et al. (2021) are interesting because pyrolysis and gasification innovators report that the unique selling point (USP) of their technologies are their versatility in processing wastes that are too complex or contaminated to undergo mechanical sorting and reprocessing, either because they are multilayered or because they are technically and or economically challenging to sort (Ragaert et al., 2017; Solis et al., 2020). However, Schwarz et al. (2021) indicate that significant sorting is required upstream of both gasification and pyrolysis of plastics where the process outputs (gas and liquid) are intended to be used as feedstock for plastics production, and this could increase the overall life cycle carbon emissions enough to nullify the potential benefits.

The study and comparisons made by Khoo (2019) and the study by Schwarz et al. (2021) provide a helpful indication of the potential life cycle carbon emissions from gasification and pyrolysis of waste plastics. However, the high uncertainty associated with LCA results, especially with technology that is barely tested at scale, means that it is challenging to draw a robust conclusion. As demonstrated by Schwarz et al. (2021), there are some highly sensitive parameters for both approaches that can weaken the environmental ‘business case’ in comparison to mechanical reprocessing. Clearly neither is a panacea. Both gasification and pyrolysis have experienced significant operational limitations, including tar removal and char disposal for the former (Benedetti et al., 2017; Lopez et al., 2018; Wolfesberger et al., 2009; Zeng et al., 2020), and high energy inputs for the latter (Crippa et al., 2019; Mayer et al., 2019; Ragaert et al., 2017; Sherwood, 2020).

Regardless of any theoretical carbon reductions from pyrolysis and gasification in comparison to mechanical reprocessing, if fugitive emissions are uncontrolled, the savings may be nullified. In gasification, the emitted CO and CH_4_ both have high-climate forcing potential and in pyrolysis, uncontrolled coal combustion emissions produce black carbon and CO_2_ that would contribute considerably to the overall process emissions. A basic Google search for pyrolysis and ‘India’ or ‘China’ for instance, brings up numerous small-scale commercial pyrolysis units aimed at processing waste tyres and household waste. Not only are these low-tech systems unlikely to incorporate air pollution control systems, but they also have the potential to be operated without any regulatory oversight or enforcement to ensure that process emissions are controlled to protect the environment and the surrounding population. FMCG companies considering processing post-consumer plastic waste using gasification or pyrolysis should refer to the European Best Available Techniques for Incineration (Neuwahl et al., 2019) that include details on process emission control and plant operation for the technologies.

### Health

#### Process emissions

Process emissions from gasification and pyrolysis have the potential to cause serious harm to health if unmanaged. The oils from pyrolysis resemble the products of crude oil and contain a range of hydrocarbons, many of which are potentially hazardous. For example, pyrolysis of plastic packaging can produce ethylbenzene, styrene, toluene and a range of polycyclic aromatic hydrocarbons (Budsaereechai et al., 2019; Miandad et al., 2019). Pyrolysis also results in the formation of gases including hydrogen, methane, ethane, ethene, propane, propene, butane and butene (Williams and Williams, 1999).

Aside from the desirable gases themselves (hydrogen and carbon monoxide), syngas from gasification also contains several hazardous substances. Though the feedstock was not stated, an example syngas ‘contaminant’ profile was provided by Block et al. (2019) and is shown in Supplemental Table S4. The syngas from packaging plastics is unlikely to contain substantial quantities of halide, dioxins and related compounds, metals or sulphur, aside from some small quantities found in glues and labels (Gerassimidou et al., 2021). However, plastics are generally co-gasified alongside biomass or as part of refuse-derived fuel (RDF)/solid-recovered fuel (SRF) from mixed waste, which may contain a vast array of materials and substances that may result in wide ranging chemical species formation.

The tar from gasification of waste can include a wide range of highly hazardous substances; however, when compared to other materials, relatively little is produced when only plastics are used as a feedstock (Block et al., 2019). Some tar is produced, and according to Robinson et al. (2016), much of this is found in the syngas itself creating a barrier to upgrade.

Tar from gasification is also found in the char at concentrations that make it unusable for upgrade. Char from pyrolysis is generally much better quality. Multiple bench-scale efforts have been made to valorise the char from both pyrolysis and gasification of waste, and in theory it can be used to make activated carbon a highly prized and versatile substance (Bernardo et al., 2010; Miandad et al., 2019). Bernardo et al. (2012) characterised char from pyrolysis of mixed plastics, biomass and tyres, finding a range of potentially toxic elements, aliphatic hydrocarbons and aromatic hydrocarbons. Although the hydrocarbons were able to be removed using sequential solvent extraction, the metals were more problematic, presenting a barrier for upgrading the char for use. Bernardo et al. (2010) also analysed char produced from a simulated mixed waste sample and determined that all samples would be classified as hazardous under Council Decision 2003/33/CE and CEWME evaluation methods for ecotoxicity. Although there is clearly potential for further use of pyrolytic chars from plastics waste, it seems likely that the barriers to upgrading will result in the material, either being combusted, either to provide heat for the process or simply to dispose of it on-site, or disposed of as hazardous waste in either a hazardous waste incinerator or hazardous waste landfill (Defra, 2013).

The range of potentially hazardous substances produced by gasification and pyrolysis is not an inherent barrier to safe operation, and there are clearly engineering solutions to controlling process emissions, as detailed by Neuwahl et al. (2019). However, these controls are costly and require a high level of technical expertise to ensure that they are implemented and maintained to remain effective. Safe operation is not guaranteed anywhere in the world, and in countries that lack sufficiently well-resourced and effective enforcement and regulation, there is a risk that process emissions from gasification and pyrolysis may not be managed according to safe standards.

#### Hazardous waste

Unless combusted on-site for disposal or energy recovery, the hazardous chars, tars and liquids produced by gasification and pyrolysis must also be treated or disposed of safely. This is important because sufficiently managed and regulated hazardous waste landfills or incineration facilities do not exist in many parts of the Global South. For instance, it is estimated that in India, 70 wt% of hazardous waste is mismanaged (Karthikeyan et al., 2018). If this is the case, then neither gasification nor pyrolysis should be considered.

#### Accidents

Lastly, as much of the growth in pyrolysis has taken place in the Global South, plants may be constructed with limited safety considerations. Several life-threatening incidents of malfunction have been reported including: an explosion at a plant in Panchkula (India) that resulted in several workers being injured, Khanty-Mansiysk (Russia) in 2012 that resulted in eight deaths, Budennovsk (Russia), Chennai (India) in 2014 that killed one and left one injured, Joensuu (Finland) in 2014 and Furth (Germany) in 1998 that both resulted large emissions of toxic gases escaping and nearby residents being evacuated (International Power Ecology Company, 2014).

## Approach 7: Co-processing in cement kilns

### Overview

The high heat and hence energy use associated with cement production has prompted producers to explore ways to reduce fossil carbon output from the sector that currently accounts for 7% of the total global anthropogenic emissions (between 2.3 and 2.6 Gt of CO_2_eq) (Hertwich, 2020; Lehne et al., 2018). One of the most widely adopted solutions is to co-combust waste, known as SRF, alongside or instead of coal (Gerassimidou et al., 2021). This practice has been adopted across Europe and where cement production facilities substituted their energy requirements with SRF, for example, at a rate of: 42% in the UK in 2015 (MPA Concrete Centre, 2017); 83% in the Netherlands and 60% in Norway (Aranda Usón et al., 2013).

SRF is produced from non-hazardous mixed waste and therefore contains a combination of both fossil (plastics, not bio-based) and biogenic waste, the latter of which is carbon neutral when combusted (Iacovidou et al., 2018). There is some evidence from industry that plastics, which have been collected for recycling, are also used to co-fire cement kilns (Brock et al., 2021; Jiao, 2020; Republic Cement, 2020), and there is also evidence that the residues from material recovery facilities are used which contain a high proportion of plastic (Fyffe et al., 2016; Saveyn et al., 2016). However, there is no indication of how prevalent the practice of co-processing plastics that have been collected for recycling is.

### Environment

As the fuel used in cement kilns is commonly fossil oil or coal (lignite), almost any other fuel source is likely to result in at least a small reduction in carbon emissions due to fugitive emissions of methane and high energy used in coal extraction. Several LCA studies have investigated potential reduction in emissions by using alternative fuels including SRF, for instance Georgiopoulou et al. (2018), Khan et al. (2020), Vermeulen et al. (2009), Bourtsalas et al. (2018) and Malijonyte et al. (2016). However, SRF is typically comprised of a complex mixture of materials, of which, for example, 68% (wt ar) are of biogenic origin such as paper, cardboard, wood and natural textiles (Séverin et al., 2010). Therefore these studies do not provide a good indication of the potential emissions from plastic packaging waste alone. GIZ-LafargeHolcim (2020) provided some indicative emission factors for different materials co-processed in cement kilns. Factors for plastics were not reported, but the fossil element of the RDF and tyres indicates a ballpark of between ~50 and 62 kgCO_2_ GJ^−1^, respectively. This suggests co-processing of plastic packaging waste might result in the same or slightly greater emissions compared to natural gas (58 kgCO_2_ GJ^−1^) and slightly less than petrol coke (95 kgCO_2_ GJ^−1^) or coal (98 kgCO_2_ GJ^−1^).

Only one review by Lazarevic et al. (2010) compared the use of plastic in cement kilns with other treatment sources, identifying just two relevant papers. The first, Shonfield (2008), presented a model that compared a range of end-of-life treatment processes for plastics waste including combustion of the plastic fraction of SRF in cement kilns. The model was theoretical, as no process data or prior publications were available for the combustion of plastics without other materials and found the purely plastic SRF combustion to provide a net carbon emissions reduction in comparison to the use of coal, despite the fact that most plastic is of fossil origin. The reason provided is that coal extraction releases fugitive emissions of methane, a small yet non-negligible source of emissions from the life cycle of coal used in combustion, reported as 1.91–4.23 g CH_4_ kg^−1^ of coal (ar) for over and underground mined coal, respectively (Spath et al., 1999).

The second study reviewed by Lazarevic et al. (2010) was by Jenseit et al. (2003), which compared the life cycle impacts of plastic used to co-fire cement kilns with landfill and conventional mechanical reprocessing for extrusion. As with Shonfield (2008), the SRF co-firing performed slightly better than incineration, but considerably worse than mechanical reprocessing, which showed the least environmental impacts overall in both studies.

Two further studies were identified here that assess the life cycle of plastics co-fired in cement kilns. The first, Schmidt et al. (2009), did not involve plastics typically used in packaging. They compared an ‘end-of-life’ scenario in which used tyres were partly used to produce artificial turf and partly used as a modifier in asphalt with a scenario where the tyres were co-combusted in a cement kiln. Overwhelmingly, the cement kiln scenario produced much higher emissions, adding confidence to the premise that mechanical reprocessing provides greater overall life cycle benefits. The second, Meys et al. (2020) compared chemical recycling, mechanical reprocessing and plastics co-fired in cement kilns. The research found replacing lignite in cement kilns provided greater benefits compared to incineration with energy recovery, chemical recycling for fuel production and feedstock, but was performing worse than chemical recycling to monomers and mechanical reprocessing. Although the comparisons with incineration and mechanical reprocessing are broadly commensurate with the other studies, the various chemical recycling pathways are less certain. As acknowledged by Meys et al. (2020), these technologies are still relatively nascent and it is likely that future learning will improve efficiencies.

### Health

Most studies on the human health risks related to atmospheric emissions from co-firing alternative materials relate to SRF produced from mixed (often residual) municipal solid waste rather than plastic waste. For instance, studies by Rovira et al. (2010, 2016) investigated environmental media near two plants in Spain, finding no notable difference in concentrations of potentially toxic elements, particulate matter and dioxins and related compounds in soil, plants and air surrounding the facilities.

In a bench-scale trial, Conesa et al. (2011) characterised emissions from simulated SRF, lignite co-combustion complimented with field tests of an operational kiln that co-combusted with SRF. Sulphur dioxide emissions decreased as a consequence of the reduced lignite and emissions of several polychlorinated dibenzo(p)dioxin and furans (PCDD/F) congeners increased very slightly compared to the reference sample (coal), but were still much lower (range: 4.42–8.48 pg I-TEQ Nm^−3^) than the permitted levels of 100 pg I-TEQ Nm^−3^. The study reported that these findings were commensurate with four other sources (not reviewed here). Conesa et al. (2011) remarked that emissions of dioxins and related compounds were more likely to come from organic material in the plant feedstock (fuel). In addition, the study found concentration changes to be negligible for the six priority pollutant polycyclic aromatic hydrocarbons, and hydrogen fluoride was not detected at all. Hydrogen chloride was slightly elevated, but within legal limits and though some small changes were detected in the levels of potentially toxic elements, they were all well within legal limits.

As a comparator, theoretical modelling carried out by Shonfield (2008) reported human toxicity potential of mixed plastic co-fired in cement kilns at approximately 1100 kg eq. dichloromethane t^−1^ plastic combusted, less than landfill and incineration, but approximately double that of all other technologies including mechanical reprocessing and pyrolysis.

The limited evidence for the emission of hazardous substances from co-firing post-consumer plastic packaging in cement kilns indicates that they are unlikely to be greater than for coal alone and that they may even be less. In Europe, facilities that co-fire waste in cement kilns are regulated at member state level by Directive 2000/76/EC (European Union, 2000a) as detailed in the section for Health under Approach 8. The Directive requires plants to be constructed and operated according to a series of best available techniques (BATs) detailed by Neuwahl et al. (2019). Cement kilns tend to be operated by large multinational corporations with sufficient resources to implement air pollution control practices. Notwithstanding this, it is possible that environmental emissions may not be managed comprehensively in less stringently regulated contexts. However, no evidence was found to support that speculation.

## Approach 8: Incineration with energy recovery

### Overview

Waste incineration with energy recovery is one of the most rapidly expanding approaches to treating mixed municipal solid waste (typically the residual part, not targeted for recycling), favoured for its effectiveness in reducing its mass (75 wt%), volume (90% v/v) and bioactivity (Christensen et al., 2011a, 2011b; Dalager et al., 2011; Hjelmar et al., 2011; Niessen, 2010). More than 500 municipal solid waste incinerators exist in Europe (Blasenbauer et al., 2020); approximately 75 in the US (United States Environmental Protection Agency, 2019), 1200 in Japan (Amemiya, 2018), 172 in Korea (Bourtsalas et al., 2019) and 390 in China (Ministry of Housing and Urban-Rural Development (MoHURD), 2019), where the approach is fast becoming the dominant form of waste treatment (Li et al., 2015).

The high cost of construction and operation in comparison to land disposal (SYSTEMIQ and The Pew Charitable Trust, 2020) has meant that incineration has not been implemented extensively in the Global South. Incinerators require considerable expertise to operate, and there have been several notable plant failures, for instance in India as reported by Nixon et al. (2017). In another example, a facility completed in 2018 by a European Chinese consortium in Addis Ababa, Ethiopia, was shut down during the early stages of commissioning. Mutethya (2020) indicated that the plant is now up and running again, though there is little further information to indicate the ongoing success of the project.

Other examples include a planned facility in Kenya (Najimesi, 2019), at least three in Delhi (Central Pollution Control Board, 2021), ‘inevitable’ planned plant construction in Malaysia (Kadir et al., 2013), at least one in Indonesia (Bahrah et al., 2020) and at least one in Myanmar (JFE Engineering Corporation, 2017).

### Environment

Studies that assess the life cycle emissions of incineration predominantly focus on the combustion of mixed municipal solid waste. These studies show lower climate forcing emissions from incineration compared to landfill across the life cycle (Laurent et al., 2014a). This is because modern incinerators almost always recover energy (EfW plants), a large portion of which comes from biogenic sources (Zheng and Suh, 2019) and because landfill generates methane, which is never captured in its entirety (Bel Hadj Ali et al., 2020).

Plastics that have been collected for recycling are rarely incinerated in isolation, resulting in a corresponding paucity of process information to determine the life cycle emissions from the practice. With the exception of Frees (2002) and perhaps one scenario reported by Shonfield (2008), incinerated plastics result in higher-climate forcing emissions compared to mechanical reprocessing (Lazarevic et al., 2010). Although approximately 1.9 Mt of bio-based plastics were produced in 2021 (European Bioplastics, 2020), 368 Mt are still made from fossil carbon (PlasticsEurope, 2021), and therefore result in an emission profile similar to fossil fuels. The small comparative benefit from incineration of waste plastic in comparison with gas and coal is that the latter two fuels result in fugitive emissions of methane during their extraction alongside the fossil fuels combusted during the process (Spath et al., 1999; Turconi et al., 2013). By comparison, the extraction of oil to produce the plastics that have become waste rests outside the system boundary used to assess the life cycle benefits of plastics combustion in incineration plants (Alhazmi et al., 2021).

### Health

Incinerators operated in the Global North have been subjected to increasingly stringent emissions thresholds since the late 1990s and early 2000s (Supplemental Table S5). For example, in Europe, emissions are strictly controlled and regulated under the Industrial Emissions Directive (European Union, 2010). Some persistent organic pollutants, polycyclic aromatic hydrocarbons, potentially toxic elements and particulate matter are still emitted, but at very low levels. For example, in the UK, particulate matter emissions from waste incineration represent just 0.02% of emissions from all sources, both nationally and in the immediate vicinity of incinerators (Maynard et al., 2010).

**Figure 1.**
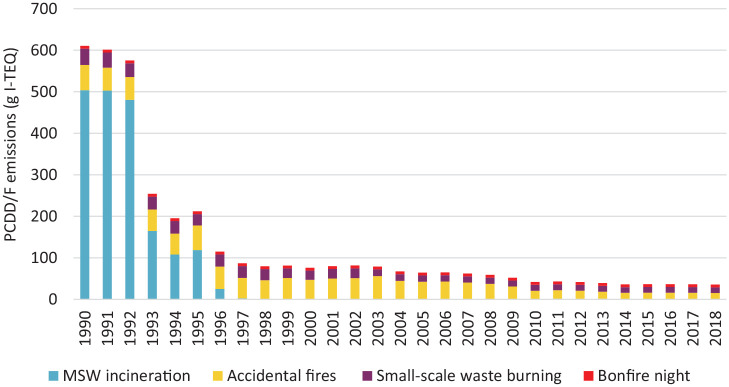
Dioxin emissions from open burning and waste incineration in the UK; data from National Atmospheric Emissions Inventory (2020). PCDD/F: polychlorinated dibenzo(p)dioxin and furans; I-TEQ: international toxic equivalency.

The effect of the Waste Incineration Directive (European Union, 2000) limits on dioxin emissions in the UK provides an indication of the capability of emissions abatement practices, which have advanced considerably (Joint Research Centre, 2018). As illustrated in Figure 1, the previously high emissions of PCDD/F from UK incinerators was reduced to negligible quantities by 1997. Since then, dioxins from waste combustion have been almost entirely generated by small-scale waste burning; accidental fires; and on November the 5th (`bonfire night’, a traditional celebration during which large fires are burned all over the UK).

Several studies indicate that the risk to human health from incineration is likely to be minimal (Douglas et al., 2017; Freni-Sterrantino et al., 2019; Ghosh et al., 2019). Although some non-negligible negative health outcomes are highlighted in systematic reviews by Tait et al. (2020) and Ashworth et al. (2014), the evidence reviewed in both studies was temporally various, and some studies were based on decades old incinerators that do not comply with current BATs.

In China, municipal solid waste incineration emissions standards have followed a step-wise pattern of improvement in much the same way as was done in European the US (Cheng et al., 2010; Ji et al., 2016). With the exception of Pb and Cd, China’s limits, outlined in GB 18485-2014, are roughly on a par with Europe’s and are more stringent for sulphur dioxide (Wu, 2018). Aside from China, there are two concerns with the implementation of waste incineration infrastructure in other parts of the Global South: (1) some countries do not have effective, independent, well-resourced enforcement and regulation to ensure that process emissions are controlled to within safe limits (SYSTEMIQ and The Pew Charitable Trust, 2020) and (2) the technology restricts the access of the informal recycling sector to valuable materials, which contribute to their income (IJgosse, 2019).

## Assessment of risk to human health and the environment when implemented in the Global South

The grouped approaches are shown in Table 6 alongside their scores for environmental and public health risk (show separately) and appropriateness for implementation in the Global South.

**Table 6.** Qualitative indication of the risk to human health, and the environment and human health from eight approaches to recovering value from post-consumer plastic packaging waste that has been collected for recycling in the Global North and South; alongside the risk of operating below environmental and health protection standards in the Global South.

Recovery type		Material recycling							Energy recovery		
Group		Group 1a: Conventional reprocessing		Group 1b: Mineral–polymer composites		Group 2: Chemical recycling			Group 3: Combustion with energy recovery		
Approach number		Approach 1	Approach 2	Approach 3		Approach 4	Approach 5	Approach 6		Approach 7	Approach 8
Approach name		Conventional mechanical reprocessing	Bottle-to-fibre mechanical reprocessing	Mineral–polymer composites: road surfacing	Mineral–polymer composites: bricks and tiles	Solvent-based purification	Chemical de-polymerisation (chemolysis)	Pyrolysis and gasification for feedstock	Pyrolysis and gasification for fuel	Co-processing in cement kilns	Incineration for energy recovery and two-stage gasification*
Global North	Environment	L	L	L	ID	ID	ID	ID	MH	MH	MH
	Human health	L	L	ID	ID	ID	ID	ID	L	L	L
Global South	Environment	ML	ML	ML	ML	ID	ID	ID	MH	MH	H
	Human health	ML	ML	ID	ID	ID	ID	ID	H	H	H
Commercial and technological maturity		H	H	MH	MH	L	L	L	MH	H	H
Risk of operating below standards in Global South (appropriateness)		ML	ML	ML	ML	H	H	H	H	H	H
Meaning of colours		Low risk/high maturity		Medium-low risk/medium-high maturity		Medium-high risk/medium-low maturity			High risk/low maturity		

* Two-stage gasification refers to the process whereby syngas is created through gasification and then *n* subsequently combusted in a downstream chamber within the same process. Qualitative scoring of risk to environment and human health and risk of operation below standards in the Global South: L: low risk; ML: medium-low risk; MH: medium-high risk; H: high risk. Qualitative scoring of level of commercial and technological maturity: L: low maturity; ML: medium-low maturity; MH: medium-high maturity; H: high maturity. Approaches are organised into Groups 1a, 1b, 2 and 3 according to the similarity of scores detailed in Section 2.4.3.

### Group 1a (conventional reprocessing)

Conventional mechanical reprocessing (Approach 1) and bottle-to-fibre mechanical reprocessing (Approach 2) are likely to result in the fewest environmental emissions and cause the last harm to public health when implemented in the Global South in comparison to other approaches. The types of plastics used in plastic packaging are unlikely to result in emissions that are harmful to workers, if adequate ventilation is provided. Although there is evidence that operators in the Global South do not always provide such control measures, they are low-cost, low-tech and easier to implement in comparison to the engineering controls required to mitigate emissions for the approaches in Groups 2 and 3. Such interventions to enable smaller, low-tech mechanical reprocessors to implement safe systems of work could be driven by FMCG companies, supporting their efforts to improve the circular economy whilst building capacity and improving working conditions in the Global South.

Little evidence was found to distinguish between the risk of harm to human health associated with Approaches 1 and 2, and the evidence for avoided life cycle burdens is comparable. Both approaches are mature, having been implemented at commercial scale since at least the 1980s and 1990s, respectively.

### Group 1b: Mineral–polymer composites

Lack of published data for the mineral–polymer composite Approaches (A3a and A3b) makes them challenging to assess. As with extrusion, emissions from the types of materials and substances used in plastic packaging are unlikely to result in a serious risk to human health, though in the case of the brick and tile production, consideration is advised that, if inhaled, the emissions from open fires could be more harmful than the emissions from the heated plastics themselves.

Black carbon produced by the open uncontrolled fires used to heat plastics has a high global warming potential that could offset some of the benefits brought about by the inferred avoided burdens of the energy-intensive conventional brick and tile production (concrete or ceramics). Similarly, the use of plastic packaging waste to modify bitumen in road surfacing is unlikely to result in more harmful emissions than those already emitted by hot bitumen during surfacing operations. Though no specific evidence was found in support, the fact that modified bitumen increases the durability of roads, infers that its use could avoid burdens associated with road resurfacing and repairs over time.

One possible concern is that plastics with potentially hazardous properties will be used as feedstock alongside the comparatively benign packaging plastics. Moreover, the risk that plastic packaging itself contains substances that are harmful when emitted during heating should not be overlooked, especially as the composition of plastics used in some parts of the Global South are not necessarily strictly controlled. It is recommended that the approaches are explored with caution until a better understanding of the potential risk from emissions is explored further.

### Group 2: Chemical recycling

Three groups of approaches exhibited very similar assessment scores in this study and were therefore grouped together for ease of discussion despite the array of technological difference between them. There is no strong evidence that any of the so-called chemical recycling techniques are commercially viable methods of producing feedstock for plastics production. As such there is also limited evidence of the associated emissions and environmental benefits beyond theoretical modelling.

The processes all involve either heat, pressure, gases or chemical solvents. Controlling these phenomena requires expertise, financial resources and ongoing maintenance to ensure that the health of workers, the public and the environment are not compromised. Moreover, the potentially hazardous solid wastes generated from some processes, such as used solvents, tars and chars from gasification and pyrolysis, and air pollution control residues, require careful handling, treatment and disposal to ensure that they do not present an ongoing environmental hazard in the future. Many countries in the Global South do not have access to landfills let alone specialist hazardous waste disposal facilities. This factor alone, is a barrier to safe and sustainable implementation of any process that produces hazardous solid waste.

### Group 3: Combustion with energy recovery

Incineration of post-consumer plastic packaging waste that has been collected with the intention to be recycled does not recover value at the material level and has a similar global warming potential to other fossil fuels; notably, such as resource recovery route cannot be accounted for as ‘recycling’ by any commonly stated definition – technical or legal (Supplemental Table S8). Incineration (with or without combined heat and power or tri-generation; and without carbon capture storage or carbon capture and utilisation) is also likely to result in higher emissions of fossil CO_2_ in comparison to all the other approaches; a gap that would possibly widen as electricity grids decarbonise over the coming decades, reducing the burdens for other technological options such as mechanical processing.

Very little information exists to evidence CO_2_eq emissions from co-processing waste plastics in cement kilns because most studies focus on SRF, a mixture of biogenic and fossil material from mixed non-hazardous waste. It is possible that across the life cycle, the use of waste plastics as a fuel in place of coal is likely to result in a marginal benefit due to the fugitive emissions during coal extraction.

Pyrolysis to fuel is a more mature approach compared to other ‘chemical recycling’ technologies and has the theoretical potential to be operated safely. In the case where pyrolysis oils are burned to generate electricity or power vehicles, the overall process may result in slightly fewer climate forcing emissions compared to other fossil fuels such as coal or oil. However, as the energy grid and vehicle fuel systems decarbonise, this benefit will dwindle.

Some studies have indicated that hot-water washing used to remove surface contamination from soiled plastic packaging may result in much higher emissions from mechanical reprocessing, in effect tipping the balance in favour of incineration. However, these comparators seem easily avoidable by (1) using sodium hydroxide in place of hot water and (2) changing the energy source for mechanical reprocessing. However, arguably these considerations do not extend on a complete environmental assessment.

## Conclusions and prospects

Our review compared eight approaches to managing post-consumer plastic packaging waste during the resource recovery phase to determine whether they are ‘appropriate’ for implementation in countries that lack an independent, well-resourced and effective environmental and safety regulator. We assessed the potential for harm to human health and the environment from each technological solution by considering its maturity alongside the costs and specialist resources that might be required to mitigate such harm.

All processes have the potential to be managed safely, but safe operation is not guaranteed anywhere. We do not suggest that waste valorisation in the Global South is inherently hazardous. However, the resources and expertise that are required to implement engineering and management safety controls vary between approaches. Wherever resources are scarce, approaches that require fewer resources to mitigate risk may be more appropriate. For example, at its most basic, brick and tile production from mineral–polymer composites (Group 1b) requires little more than a mould, a trowel and a metal container on an open fire. Respiratory inhalation of emissions from the fire and melting plastic has been highlighted as a risk, but the hazards are easily and cheaply surmountable with basic engineering controls such as personal respiratory protection equipment and careful source management of feedstock. For technologies, such as incineration (Group 3) and chemical recycling (Group 2), safety management is more challenging, partly not only because of the high heat and pressure involved, but also because many of the chemical and material outputs can be highly hazardous to human health and the environment. Most countries in the Global South still lack comprehensive waste management for non-hazardous waste, let alone specialist facilities for managing such hazardous by-products. Conventional mechanical reprocessing operations (Group 1a) are mature and capable of handling large amounts of material. Although there are hazards involved with both small and large operations, the expertise and cost to control those hazards is minimal in comparison to incineration or co-processing in cement kilns.

Our review focused on the final stages of the waste material flow that involve reprocessing and recovery, touching briefly on the risk involved with secondary use of mechanically reprocessed plastics. We did not consider in detail the wider waste system, in particular the systems that involve collection, sorting and supply, much of which is carried out by large numbers of informal actors, waste pickers who operate outside of mainstream waste management planning, often without and safe systems of work. Informality or not, we did not address the dilemma between small-scale, decentralised approaches and large scale, high-throughput processing (economies of scale). Even if affordable, implementing safety and environmental protection measures is a substantial challenge across the predominantly informal waste sector in the Global South. However, to exclude informal actors in favour of large scale, on the basis of Western standards of safe operation, could risk cutting off material supplies whilst restricting income generation potential of some of the world’s poorest people.

Chemical recycling processes have received attention in recent years for their potential to handle material that is either highly soiled, bonded into an assembly or bonded into a multilayer composite. Yet the evidence indicates that none of these USPs have been realised, and no evidence was found to indicate that chemical feedstock is being produced from post-consumer plastic packaging in a commercially sustainable process.

Innovation of new processes must be supported, but the urgency with which action must be taken to improve our use of resources and reduce plastic pollution is a much greater concern. Mechanical reprocessing is both commercially mature and has the lowest environmental impact compared to all the other approaches reviewed here. It is already implemented worldwide: as a highly mechanised process in the Global North and as a more labour-intensive operation in the Global South. The principle limitation of mechanical reprocessing is that it is unsuitable for processing multilayer (multi-polymer) materials. In response, the hope that chemical recycling will actualise is proffered. Given how unlikely this is in the near future, it is suggested that product redesign is considered.

Though some of the approaches were reasonably well-evidenced, process data for operations in the Global South is very limited or non-existent and therefore not easily discoverable for most processes. Our review was comprehensive but not exhaustive. Only a carefully conducted systematic review will reveal the true state of evidence on this topic, and it is strongly recommended that such a process is carried out, building on our work and bringing a more robust insight into the topic.

## Supplemental Material

sj-pdf-1-wmr-10.1177_0734242X221105415 – Supplemental material for Scaling up resource recovery of plastics in the emergent circular economy to prevent plastic pollution: Assessment of risks to health and safety in the Global South Click here for additional data file. Supplemental material, sj-pdf-1-wmr-10.1177_0734242X221105415 for Scaling up resource recovery of plastics in the emergent circular economy to prevent plastic pollution: Assessment of risks to health and safety in the Global South by Ed Cook, Costas A Velis and Joshua W Cottom in Waste Management & Research
