# Supplementary material for: Scaling up resource recovery of plastics in the emergent circular economy to prevent plastic pollution: Assessment of risks to health and safety in the Global South
Source: Waste Manag Res. 2022 Jul 23;40(12):1680–707. doi: 10.1177/0734242X221105415 (PMC9606178; doi:10.1177/0734242X221105415)
Supplement: sj-pdf-1-wmr-10.1177_0734242X221105415 – Supplemental material for Scaling up resource recovery of plastics in the emergent circular economy to prevent plastic pollution: Assessment of risks to health and safety in the Global South [file sj-pdf-1-wmr-10.1177_0734242X221105415.pdf]

Supplemental Information

Scaling up resource recovery of plastics in the emergent circular economy to prevent plastic pollution: Assessment of risks to health and safety in the Global South

https://doi.org/10.1177/0734242X221105415

Ed Cook[a], Costas A. Velis[a,*], Joshua W. Cottom[a]

[a]*School of Civil Engineering, University of Leeds, Leeds, LS2 9JT, UK*

*Corresponding author: c.velis@leeds.ac.uk; Telephone: +44 (0) 113 3432327; Room 304, School of Civil Engineering, University of Leeds, Leeds, LS2 9JT, United Kingdom

Keywords: Plastic, solid waste, health and safety, Global South, resource recovery, circular economy.

Here we present information on: i) Criteria for assessing commercial and technological maturity (**Table S1**); (ii) Water consumption and wastewater used in mechanical reprocessing (**Table S2**); iii) Multimedia evidence review for brick and tile production in the Global South; iv) Contaminants found in syngas (**Table S4**); v) Comparison of emission thresholds for incineration (**Table S5**); vi) Summary of environmental and health risks for each of the eight approaches (**Table S6**); vii) Scoring of technological and commercial maturity (**Table S7**); and viii) International definitions of recycling (**Table S8**).

Table S1: Criteria for assessing commercial and technological maturity.

Rank	Description
Low maturity	Technology is still on the technological readiness level (TRL) scale as described by the U.S. Department of Energy (2011). It may be at TRL 9, having proved it can operate under the full range of expected conditions, but there is limited evidence of commercially sustainable implementation.
Medium-low maturity	Evidence of commercially sustainable implementation but either at small scale or there are doubts about commercial viability – approach may be subsisting on innovation funding.
Medium-high maturity	Evidence of commercially sustainable implementation, but has yet to reach widespread prevalence as a preferred approach to treatment or may not be scalable.
High maturity	Approach is prevalent, mature and has been implemented at scale for many years or decades.

Table S2: Water consumption and waste-water discharge (L t^{-1} material processed) at plastics reprocessing facilities in China; after Chen et al. (2019).

Categories	Substances	PP	PE	PVC	ABS	PS	PET
Consumption	Industrial water	420	388	453	361	340	372
Discharge	Waste water	302	286	372	338	227	240

Table S3: Summary of practices involving the production of mineral-polymer composite paving and tiles in the Global South observed on multimedia posts.

Source	Context	Feedstock	Process description	Dry/ wet	Soph. cat.	Hazards 1	2	3	4	5	6	7	8	Mitigation measures or comments
Kolev (2019)	Bulgaria	Unknown plastic scrap	Comminuted material is mixed with sand and heated in semi-continuous / semi- manual batch process					x				x		• Demonstration plant appears to have basic safe system of work though shorts
	Russia	unknown plastic	Planetary Ecologist: Tile production with and sand which are ‘extruded’ together and pressed in batch es in a hot press	Dry	low			x					x	• No obvious safe system of work • No personal protective equipment used
	Pune India	unknown plastic	Andrey Kolev: Low speed high torque cutting mill with plastic and crushed rock together and then forced into a piston press extruder pressed into mould by hand	Dry	Med–low		x	x						• Exhaust treatment phase noted by narrator
	Uganda	WEEE and ELV plastics	Zecoplast Recycling: River sand used with and comminuted and dried then added to a batch furnace- batch es removed by hand to produce roof tiles	Dry	med							x	x	• Gloves and face masks used but many on foreheads • Appears to be a safe system of work in place though serious concern about the processing of end of life vehicle and waste electrical and electronic equipment plastics
Earth Titan (2019)	Philippines	unknown plastic	Florence Blondel: Batch hot is gravity fed mix into mould mixer then pressed by hand	Dry	low			x						
NTVUganda (2013)	Uganda	HDPE durable household goods (ELV)	Resintile EA Ltd. Kiln dried sand is sieved and mixed with comminuted plastic fragments	Dry	Med			x				x		• General evidence of safe system of work including use of personal protective equipment • Concern over the processing of end of life vehicle and waste electrical and electronic equipment plastics that were mentioned by the manager

Hazard codes as follows: 1) Unguarded fast or high torque machinery in close proximity to workers; 2) Worker interaction with machinery resulting in risk of being drawn in; 3) High temperature equipment in close proximity to workers risking burns; 4) Risk of interaction with unknown potentially hazardous materials or substances; 5) Risk of burns from caustic substance; 6) Particle loss to the environment likely; 7) Risk of aerosolised hazardous substance; 8) Risk of ballistic injury to hands, feet, body from interaction with sharp or heavy objects.

Table S4: Main syngas contaminants with their emissions in waste gasification and the target levels of the major applications; after Block et al. (2019).

**Contaminant mg Nm^{-3}**	**Waste gasification**	**Gas engine**	**Gas turbine**	**Methanol synthesis**	**Fischer- Tropsch synthesis**	**EU emissions standards[a]**
Particulates	10^4-10^5	< 50	< 5	< 0.02	n.d.	10
Tar	0-20,000	< 100	< 10	< 0.1	< 0.01	n.s.
Sulphur (H_2S, COS)	50-100	< 20	< 1	< 1	< 0.01	50 (SO*)
Nitrogen (NH_3, HCN)	200-2000	< 55	< 50	< 0.1	< 0.02	200 (NO_x)
Alkali metals	0.5-5	n.s.	< 0.2	< 0.2	< 0.01	n.s.
Halides (HCl)	0-300	< 1	< 1	< 0.1	< 0.01	10
Heavy metals	0.005-10	n.s.	n.s.	n.s.	< 0.001	0.03 (Hg)
Dioxins/furans ng-TEQ/m^3	n.s.	n.s.	n.s.	n.s.	n.s.	0.1

[a]At 11% O_2. Abbreviations Not detectable (n.d.); not specified (n.s.). Fischer-trope synthesis converts CO and H_2 into liquid hydrocarbons in the presence of catalysts.

Table S5: Comparison of emission thresholds for incineration in different jurisdictions; after Cheng and Hu (2010) except GB 18485-2014 which was after Wu (2018).

		**China[a] (GB 18485-2001)**		**China (GB 18485-2014)**		**European Union[b]**		**United States**			
**Pollutant**	**Units**							**Small MSW combustor[d]**		**Large MSW combustor[h]**	
PM	mg m^{-3}	80	(Hourly ave.)	30	(Hourly ave.)	10<br>30	(Daily ave.);<br>(Half-hourly ave.)	24	(3-run ave.)	25<br>20	(existing unit)<br>(new unit)
Smoke opacity	mg m^{-3}	20%	(Hourly ave.)		(Hourly ave.)	-		10%	(Hourly ave.)	-	-
CO	mg m^{-3}	150	(Hourly ave.)	100	(Hourly ave.)	50<br>100	(Daily ave.)<br>(Half-hourly ave.)	50-150	(ppm by volume)[e]	50-250	(ppm by volume)[i]
SO_2	mg m^{-3}	260	(Hourly ave.)	100	(Hourly ave.)	50<br>200	(Daily ave.)<br>(half-hourly ave.)	30	(ppm by volume, daily block geometric ave.)	29<br>30	(ppm by volume, existing unit);<br>(ppm by volume, new unit)
NOx	mg m^{-3}	400	(Hourly ave.)	300	(Hourly ave.)	200 or 400<br>400	(Daily ave.)[c]<br>(Half-hourly ave.)	150 or 500	(ppm by volume, daily block arithmetic ave.)[f]	165-250<br>180 or 150	(ppm by volume, existing unit)<br>(ppm by volume, new unit)[j]
HCl	mg m^{-3}	75	(Hourly ave.)	60	(Hourly ave.)	10<br>60	(daily ave.)<br>(half-hourly ave.)	25	(ppm by volume, 3-run ave.)	29<br>25	(ppm by volume, existing unit)<br>(ppm by volume, new unit)
Hg	mg m^{-3}	0.2		0.05		0.05	(measured ave.)	0.08		0.05	
Cd	mg m^{-3}	0.1		0.1		0.05	(sum of Cd & Tl)	0.02		0.035<br>0.01	(existing unit)<br>(new unit)
Pb	mg m^{-3}	1.6		1		0.5	(sum of Sb, As, Pb, Cr, Co, Cu, Mn, Ni, and V)	0.2		0.4<br>0.14	(existing unit)<br>(new unit)
Dioxins/ furans	ng TEQ m^{-3}	1		0.1		0.1		13	(total mass basis)[g]	30 or 35<br>13	(existing unit, total mass basis)[k]<br>(new unit, total mass basis)

Notes: [a] all emission limits except opacity are measured at 11% oxygen, dry basis at standard conditions (State Environmental Protection Administration of China, 2001a); [b] all emission limits are measured at 10% oxygen, dry basis at standard conditions (European Union, 2000); [c] the daily average limit is 200 mg m^{-3} for new and existing plants with more than six tonnes per hour capacity, and 400 mg m^{-3} for existing plants with no more than six tonnes per hour capacity; [d] for unit with an individual municipal waste combustion capacity of 250 tonnes per day or less, all emission limits except opacity are measured at 7% oxygen, dry basis at standard conditions (U.S. Environmental Protection Agency, 2000); [e] the limit varies by combustor technology; [f] the limit is 150 ppm by volume for Class I units (located at municipal waste combustion plants with an aggregate plant combustion capacity more than 250 tonnes per day of MSW) or 500 ppm by volume for Class II units (located at municipal waste combustion plants with an aggregate plant combustion capacity no more than 250 tons per day of MSW); [g] dioxins/furans on total mass basis measured as tetra- through octachlorinated dibenzo-p-dioxins and dibenzofurans. Not toxic equivalent (TEQ) value; [h] for unit with an individual municipal waste combustion capacity of greater than 250 tonnes per day, all emission limits except opacity are measured at 7% oxygen, dry basis at standard conditions (U.S. Environmental Protection Agency, 2006c); [i] the limit varies by combustor technology; [j] the limit varies by combustor type for existing unit, while for new unit it is 180 ppm by volume in the first year of operation and 150 ppm by volume after first year of operation; [k] the limit is 30 ng/m_3 for non-electrostatic precipitator (ESP) equipped unit or 35 ng m^{-3} for ESP-equipped unit.

Table S6: Summary of environmental and health impacts from approaches to managing plastic packaging waste in the Global North and South.

		Global North		Global South	
	Approach	Environment	Health	Environment (additional)	Health (additional)
1	Conventional mechanical reprocessing for extrusion	• Lower carbon emissions than most other processes, particularly waste to energy incineration and SRF cement kiln incineration (Lazarevic et al., 2010; Bernardo et al., 2016)<br>• Hot-water washing has the potential to increase overall life-cycle emissions, though the evidence to support this assertion is limited (Krogh et al., 2001; Frees, 2002)<br>• If waste-water discharge is controlled and managed, both debris and biochemical emissions are likely or have the potential to be minimal (Lassen et al., 2015; Cole and Sherrington, 2016; Boucher and Friot, 2017; Operation Clean Sweep, 2020)	• Emissions from extrusion of packaging plastics are generally well regulated and controlled through a variety of mechanical processes alongside duty of care systems to establish material provenance (Unwin et al., 2013; Cook et al., 2020)<br>• Workplace hazard management is broadly aligned with other sectors	• In some cases, carbon emissions may be lower due to greater manual processing in comparison to the Global North<br>• There is some evidence that coal combustion is used to generate heat (Aryan et al., 2019) – potentially cancelling out any benefit<br>• Water containing biological material washed from the surface of plastic has the potential to harm aquatic environments if not treated (Aryan et al., 2019)<br>• Waste-water discharge may not always be controlled resulting in pellet an fragment loss to surface and foul-water drainage (**Table 4**)	• Evidence of lack of management for atmospheric emissions from extrusion and lack of respiratory protective equipment (**Table 4**)<br>• Evidence of workers being exposed to a variety of workplace hazards including risk of being drawn in to fast-moving or high-torque machinery, contact with hot machinery and lack of PPE (**Table 4**)
2	Bottle-to-fibre reprocessing for extrusion	• As with conventional mechanical reprocessing for extrusion, this is mature technology that is likely to have similarly low carbon emissions compared to thermal processes as the displacement energy is broadly similar (RDC-Environment, 2010; Shen et al., 2011; Komly et al., 2012)<br>• Little data exists to indicate water usage from fibre spinning though objective reasoning suggests it is largely the same as bottle-to-bottle (**Section 4.2.2**)	• Health implications are considered similar to mechanical recycling although the use of only one polymer (PET) that is mainly used in packaging may lower the risk of contamination from materials that have been used in other applications, for instance end of life vehicles or electrical equipment (see above)	• As conventional mechanical reprocessing for extrusion (see above)	• As conventional mechanical reprocessing for extrusion (see above)
3a	Mineral-fibre composites: roads	• Objective reasoning suggests that increased durability of polymer modified surfaces will reduce the need for replacement inferring a strong case for the use of plastics in these applications<br>• Risk of plastic particle emissions to the environment exists though there is virtually no empirical data to help determine the magnitude – one study reports it as likely minimal and due mainly to studded tyres used in low temperature climates (Rødland, 2019; Vogelsang et al., 2020)	• Very little evidence for health implications however objective reasoning suggests it is broadly similar to plastics extrusion albeit at lower temperatures (Tsai et al., 2009; Yamashita et al., 2009; He et al., 2015)	• Although there is limited evidence to suggest it might happen, the potential for plastics being added to road surfaces as a method of disposal rather than to enhance durability should be considered. In such a scenario, the overall life-cycle case for this approach and result in increased particle emissions	• Very little evidence for health implications however objective reasoning suggests it is broadly similar to plastics extrusion albeit at lower temperatures<br>• Evidence of polymers combusting briefly in tile making (Kumi-Larbi Jnr, personal communication 10 December 2020) which may also occur in asphalt formulations, resulting in workers being exposed to hazardous substances
3b	Mineral-fibre composites: bricks & tiles	• n/a	• n/a	• Objective reasoning suggests a strong lifecycle benefit as a consequence of avoided concrete or ceramic production and use of otherwise wasted resources<br>• No evidence for microplastic production exists and it is recommended that this is investigated<br>• Black carbon emissions from open fire combustion may counteract any avoided burdens gained from off-setting concrete production	• Evidence of polymers combusting briefly and resulting in workers being exposed to hazardous substances emitted into the atmosphere (Kumi-Larbi Jnr, personal communication 10 December 2020)<br>• Melt formulation may also emit hazardous substances though there is limited information to evidence

	Approach	Global North		Global South	
		Environment	Health	Environment (additional)	Health (additional)
4	Solvent based purification	• This technology is not mature enough to assess	• This technology is not mature enough to assess	• This technology is not mature enough to assess	• Though this technology is far from commercialisation, it is likely that health effects will be related to the handling and discard of solvents used in the processes
5	Chemical depolymerisation	• The small number of studies available indicate that depolymerisation of ethylene glycol to produce PET starting materials results in approximately similar emissions to mechanical recycling (Shen et al., 2010; Meys et al., 2020) However the technology is only appropriate for post-industrial feedstocks and therefore unlikely to be of relevance to FMGCs at present.	• This technology is not mature enough to assess	• This technology is not mature enough to assess	• This technology is not mature enough to assess
6	Pyrolysis & gasification	• Though pyrolysis and gasification technologies are maturing, they are generally used for fuel production where the lifecycle emissions are greater than mechanical recycling but fewer than incineration with energy recovery (Khoo, 2019) (Schwarz et al., 2021)	• The outputs from these processes are mostly hazardous to human health and potentially fatal with low exposure. They should be carefully controlled to ensure that workers and the public are protected from exposure (Williams and Williams, 1999; Block et al., 2019; Budsaereechai et al., 2019; Miandad et al., 2019)	• The potential for fugitive emissions from both gasification and pyrolysis may negate any lifecycle emission savings as a result of these technologies	• In addition to controlling emissions from the processing of feedstock, local emissions from heat generation by coal, oil and recirculated gasses may result in the production of substances that may be harmful to human health (Block et al., 2019)<br>• The outputs of both of these processes require stringent control and regulatory oversight to ensure that they are handled safely. In particular the residues (char and tar) from these processes contain highly hazardous substances that would require a full duty of care system to ensure that they are treated or disposed of in a way that does not result in future harm to human health and the environment (Wolfesberger et al., 2009; Benedetti et al., 2017; Lopez et al., 2018; Zeng et al., 2020)
7	Co-processing in cement kilns	• The evidence for lifecycle emission from co-firing plastic packaging in cement kilns is limited, though strongly driven by the avoided burdens and fugitive methane emissions during coal extraction (Spath et al., 1999)<br>• However the limited data indicate it is not different to incineration with energy recovery and worse than conventional mechanical recycling (Jenseit et al., 2003; Shonfield, 2008; Schmidt et al., 2009; Lazarevic et al., 2010; Meys et al., 2020)	• Emissions from cement kilns in the Global North are managed by managing the process parameters, the feedstock com position and using air pollution control technology	• As Global North	• No data was identified to evidence emissions from cement kiln co-firing with post-consumer plastic packaging waste in the Global South. However the risk of operating in jurisdictions where insufficiently resourced environmental regulation and enforcement should be considered

	Global North		Global South	
Approach	Environment	Health	Environment (additional)	Health (additional)
8 Incineration	• Lifecycle carbon emissions are generally greater than for mechanical recycling (Shonfield, 2008; Laurent et al., 2014; Zheng and Suh, 2019; Bel Hadj Ali et al., 2020), though the impact of hot-water washing of biological surface contamination may tip the scales in favour of incineration in some circumstances Frees (2002)<br>• LCAs are strongly dependent on the energy mix in the country where implemented, therefore as decarbonisation progresses, the case for incinerating plastics is likely to diminish	• Hazardous emissions are generally minimal in well managed European incinerators (Douglas et al., 2017; Freni-Sterrantino et al., 2019; Ghosh et al., 2019), though there is some non-negligible evidence of harm to human health in one or two studies (Ashworth et al., 2014; Tait et al., 2019)	• The use of waste heat generated by incinerators in the Global South isn't well reported which may affect the life-cycle justification for their use	• Emission control limit concentrations are becoming increasingly stringent in some countries (e.g. China) and comparable to European standards<br>• There are serious concerns that emissions may not be managed, that regulation may not exists in some countries and that where it does exists it will not be enforced

Table S7: Rationale for scoring commercial and technological maturity.

Approach		Rationale for scoring	Commercial & technological maturity
1	Conventional mechanical reprocessing	History of large scale commercial operation in Global South back to at least the 1980s or 1990s	H
2	Bottle-to-fibre mechanical reprocessing	Commercial operation at scale since 1990s and currently widespread	H
3a	Mineral-polymer composites: road surfacing	Virgin plastics have been used in this application since 1980s and the technique is now widespread. However, waste has only been used very recently	MH
3b	Mineral-polymer composites: bricks & tiles	Relatively nascent technology, but proven at small scale	MH
4	Solvent based purification	Nascent and unproven commercially	L
5	Chemical de-polymerization (Chemolysis)	Nascent and unproven commercially for post-consumer packaging	L
6	Gasification for feedstock	Though the technology has existed for a long time, there is little evidence that it is a commercially mature process when applied to post-consumer waste plastic packaging where the outputs are starting materials for plastics production	L
6	Pyrolysis for feedstock	Technology is mature but has data on commercial maturity for process that uses post-consumer waste plastics as feedstock to create starting materials for plastic production	L
6	Pyrolysis & gasification for fuel	Technology is proven for production of fuel but still not heavily commercialised	MH
7	Incineration cement kiln	Solid recovered fuel co-processing is well established and although not commonly applied to waste plastics is mature enough to be transferrable	H
8	Incineration & gasification for energy recovery	Concept has existed for centuries but was heavily pollution up until the 1990s across the Global North. Since developments in air pollution control technology, around the same time, safe operation is mature	H

Scoring of maturity: low (L); medium low (ML); medium high (MH); high (H).

Table S8: Definitions of recycling and recyclable from various sources; adapted and updated after American Institute for Packaging and the Environment (2018).

Source	Standard / law	Title	Definition
International Organization for Standardization (2016)	ISO 14021:2016	Environmental labels and declarations - self-declared environmental claims (Type II environmental labelling)	'Recyclable'<br>A characteristic of a product, packaging or associated component that can be diverted from the waste stream through available processes and programmes and can be collected, processed and returned to use in the form of raw materials or products.
International Organization for Standardization (2013)	ISO 18604:2013	Packaging and the environment - Material recycling	'Material recycling'<br>reprocessing, by means of a manufacturing process, of a used packaging material into a product, a component incorporated into a product, or a secondary (recycled) raw material; excluding energy recovery and the use of the product as a fuel.
The Association of Plastics Recyclers (nd)	n/a	Recyclable per APR Definition	'Recyclable'<br>These criteria must all be met for a package to be considered "Recyclable per APR Definition".<br>At least 60% of consumers or communities have access to a collection system that accepts the item per the U.S. Federal Trade Commission "Green Guides".<br>The item must have market value, or be supported by a legislatively mandated program.<br>The item is most likely sorted correctly into a market-ready bale of a particular plastic meeting industry standard specifications, through commonly used material recovery systems, including single-stream and dual stream MRFs, PRF's, systems that handle deposit system containers, grocery store rigid plastic and film collection systems.<br>The item can be further processed through a typical recycling process cost effectively into a postconsumer plastic feedstock suitable for use in identifiable new products.
United States Environmental Protection Agency (nd)	n/a	Definitions: Utilized in the Re-TRAC Connect™ State Measurement Template	'Recycling'<br>…refers to the series of activities by which discarded materials are collected, sorted, processed, and converted into raw material and returned to the economic mainstream by being used in the production of new products. Does not include the use of these materials as a fuel substitute or for energy production (Modification of U.S. EPA 1997).
European Commission (2008)	Directive 2008/98/EC	Directive 2008/98/EC of the European Parliament and of the Council of 19 November 2008 on waste and repealing certain Directives (aka The Waste Framework Directive)	'Recycling'<br>…means any recovery operation by which waste materials are reprocessed into products, materials or substances whether for the original or other purposes. It includes the reprocessing of organic material but does not include energy recovery and the reprocessing into materials that are to be used as fuels or for backfilling operations.
Ellen MacArthur Foundation (2020)	n/a	New Plastics Economy Global Commitment: Commitments, vision and definitions	'Material recycling'<br>(see ISO 18604:2013) … includes both mechanical (maintaining polymer structure) and chemical (breaking down polymer structure into more basic building blocks, e.g. via chemical or enzymatic processes) recycling processes explicitly excludes technologies that do not reprocess materials back into materials but instead into fuels or energy.

Source	Standard / law	Title	Definition
Federal Trade Commission (2012)	Vol. 77 No. 197. 16 CFR. Part 260. pp 62122-62132	Guides for the Use of Environmental Marketing Claims; Final Rule	‘Recyclable’<br>A product should not be labelled as ‘recyclable’ — even if it is technically capable of being recycled — if it is unlikely that the product will be recycled in its ordinary usage (e.g., a trash bag). If any component limits the ability to recycle of an attribute, such as shape or size, a recyclable claim would be deceptive.<br>A product or package should not be marketed as recyclable unless it can be collected, separated, or otherwise recovered from the waste stream through an established recycling program for reuse or use in manufacturing or assembling another item. When recycling facilities are available to a substantial majority of consumers or communities where the item is sold, marketers can make unqualified recyclable claims. The term ‘substantial majority’ as used in this context means at least 60 percent. If recycling facilities are not available to a ‘substantial majority’ of consumers or communities can add qualifications clarifying facility availability.<br>Marketers can make unqualified recyclable claims for a product or package if the entire product or package, excluding minor incidental components, is recyclable. ISO 18604: 2013 Characteristic of a product, packaging, or associated component that can be diverted.

CRediT author statement

Ed Cook: Conceptualisation, methodology, investigation, writing – original and final draft; Costas A Velis: Review & editing, writing – final draft; Josh Cottom: Review & editing, writing – final draft.

Acknowledgements

We are grateful to the project advisory board and technical reviewers who provided comments and insightful feedback suggestions on earlier versions of this review, in particular: Professor David C Wilson (Independent consultant); Professor Linda Godfrey (Council for Scientific and Industrial Research – CSIR); David Lerpiniere (Resource Futures); Joanne Green (Tearfund); Mari Williams (Tearfund); Richard Gower (Tearfund). We would also like to thank the following people who kindly provided their time, advice, experience and expertise: Alexander Kumi-Larbi Jnr. (Imperial College London); Zoë Lenkiewicz (WasteAid); Sarah Edmondson; Carla Valle-Klann (UN Environment).

Funding

This research is the result of an independent review commissioned by Tearfund and funded jointly by Tearfund and the Norwegian Agency for Development Cooperation (NORAD) under Project Agreement QZA-20/0114 ('From Trash to Cash: Turning plastic pollution into economic opportunity').

Data statement

All data considered in this review can be found in the figures, tables and narrative presented in the main manuscript and supporting information.

References

American Institute for Packaging and the Environment (2018). "Packaging Materials Management Definitions: A Review of Varying Global Standards Guidance Document".).

Aryan, Y., Yadav, P., and Samadder, S.R. (2019). Life Cycle Assessment of the existing and proposed plastic waste management options in India: A case study. *Journal of cleaner production* 211**,** 1268-1283. doi: 10.1016/j.jclepro.2018.11.236.

Ashworth, D.C., Elliott, P., and Toledano, M.B. (2014). Waste incineration and adverse birth and neonatal outcomes: a systematic review. *Environment International* 69**,** 120-132. doi: https://doi.org/10.1016/j.envint.2014.04.003.

Bel Hadj Ali, N., Abichou, T., and Green, R. (2020). Comparing estimates of fugitive landfill methane emissions using inverse plume modeling obtained with Surface Emission Monitoring (SEM), Drone Emission Monitoring (DEM), and Downwind Plume Emission Monitoring (DWPEM). *J Air Waste Manag Assoc* 70(4)**,** 410-424. doi: 10.1080/10962247.2020.1728423.

Benedetti, V., Patuzzi, F., and Baratieri, M. (2017). Gasification Char as a Potential Substitute of Activated Carbon in Adsorption Applications. *Energy Procedia* 105**,** 712-717. doi: https://doi.org/10.1016/j.egypro.2017.03.380.

Bernardo, C.A., Simões, C.L., and Pinto, L.M.C. (2016). Environmental and economic life cycle analysis of plastic waste management options. A review. *AIP Conference Proceedings* 1779(1)**,** 140001. doi: 10.1063/1.4965581.

Block, C., Ephraim, A., Weiss-Hortala, E., Minh, D.P., Nzihou, A., and Vandecasteele, C. (2019). Co-pyrogasification of Plastics and Biomass, a Review. *Waste and Biomass Valorization* 10(3)**,** 483-509. doi: 10.1007/s12649-018-0219-8.

Boucher, J., and Friot, D. (2017). "Primary Microplastics in the Oceans: a Global Evaluation of Sources". (Gland, Switzerland).

Budsaereechai, S., Hunt, A.J., and Ngernyen, Y. (2019). Catalytic pyrolysis of plastic waste for the production of liquid fuels for engines. *RSC Advances* 9(10)**,** 5844-5857. doi: 10.1039/C8RA10058F.

Chen, Y., Cui, Z., Cui, X., Liu, W., Wang, X., Li, X., et al. (2019). Life cycle assessment of end-of-life treatments of waste plastics in China. *Resources, Conservation and Recycling* 146**,** 348-357. doi: https://doi.org/10.1016/j.resconrec.2019.03.011.

Cheng, H., and Hu, Y. (2010). Municipal solid waste (MSW) as a renewable source of energy: Current and future practices in China. *Bioresource Technology* 101(11)**,** 3816-3824. doi: https://doi.org/10.1016/j.biortech.2010.01.040.

Cole, G., and Sherrington, C. (2016). "Study to quantify pellet emissions in the UK". (Bristol, UK).

Cook, E., Velis, C.A., and Derks, M. (2020). Plastic waste reprocessing for circular economy: A systematic review of risks to occupational and public health from legacy substances and extrusion. *engrXiv [Preprint]*. doi: https://doi.org/10.31224/osf.io/yxb5u.

Douglas, P., Freni-Sterrantino, A., Leal Sanchez, M., Ashworth, D.C., Ghosh, R.E., Fecht, D., et al. (2017). Estimating particulate exposure from modern municipal waste incinerators in Great Britain. *Environmental Science & Technology* 51(13)**,** 7511-7519. doi: 10.1021/acs.est.6b06478.

Earth Titan (2019). "6 Roof and Pavement Tiles from Plastic Waste".).

Ellen MacArthur Foundation (2020). "New Plastics Economy Global Commitment: Commitments, vision and definitions". (Cowes, UK).

European Commission (2008). "Directive 2008/98/EC of the European Parliament and of the Council of 19 November 2008 on waste and repealing certain Directives". Official Journal of the European Union).

Federal Trade Commission (2012). "Guides for the Use of Environmental Marketing Claims; Final Rule. Vol. 77 No. 197. 16 CFR. Part 260. pp 62122-62132".).

Frees, N. (2002). "Miljømæssige fordele og ulemper ved genvinding af plast: Eksempler med udgangspunkt i konkrete produkter (in Danish)". Institut for Produktudvikling).

Freni-Sterrantino, A., Ghosh, R.E., Fecht, D., Toledano, M.B., Elliott, P., Hansell, A.L., et al. (2019). Bayesian spatial modelling for quasi-experimental designs: An interrupted time series study of the opening of Municipal Waste Incinerators in relation to infant mortality and sex ratio. *Environment International* 128**,** 109-115. doi: https://doi.org/10.1016/j.envint.2019.04.009.

Ghosh, R.E., Freni-Sterrantino, A., Douglas, P., Parkes, B., Fecht, D., de Hoogh, K., et al. (2019). Fetal growth, stillbirth, infant mortality and other birth outcomes near UK municipal waste incinerators; retrospective population based cohort and case-control study. *Environment International* 122**,** 151-158. doi: https://doi.org/10.1016/j.envint.2018.10.060.

He, Z., Li, G., Chen, J., Huang, Y., An, T., and Zhang, C. (2015). Pollution characteristics and health risk assessment of volatile organic compounds emitted from different plastic solid waste recycling workshops. *Environment International* 77**,** 85-94. doi: 10.1016/j.envint.2015.01.004.

International Organization for Standardization (2013). "Packaging and the environment - material recycling (ISO standard number: 18604)". (Geneva, Switzerland: International Organization for Standardization [ISO],).

International Organization for Standardization (2016). "Environmental labels and declarations - self-declared environmental claims (Type II environmental labelling) (ISO standard number 14021)". (Geneva, Switzerland: International Organization for Standardization [ISO],).

Jenseit, W., Stahl, H., Wollny, V., and Wittlinger, R. (2003). "Recovery options for plastic parts from end-of-life vehicles: an eco-efficiency assesment". (Brussels, Belgium).

Khoo, H.H. (2019). LCA of plastic waste recovery into recycled materials, energy and fuels in Singapore. *Resources, Conservation and Recycling* 145**,** 67-77. doi: https://doi.org/10.1016/j.resconrec.2019.02.010.

Kolev, A. (2019). "Plastic sand tile machine - only from recycling plastic and sand". (Bulgaria).

Komly, C.-E., Azzaro-Pantel, C., Hubert, A., Pibouleau, L., and Archambault, V. (2012). Multiobjective waste management optimization strategy coupling life cycle assessment and genetic algorithms: Application to PET bottles. *Resources, Conservation and Recycling* 69**,** 66-81. doi: https://doi.org/10.1016/j.resconrec.2012.08.008.

Krogh, L.v., Raadal, H.L., and Hanssen, O.J. (2001). "Life Cycle Assessment of Different Scenarios for Waste Treatment of a Plastic Bottle Used for Food Packaging: Summary".).

Kumi-Larbi Jnr, A. (personal communication 10 December 2020). *RE: LDPE Emissions discussion.* Type to E. Cook.

Lassen, C., Hansen, S.F., Magnusson, K., Hartmann, N.B., Jensen, P.R., Nielsen, T.G., et al. (2015). "Microplastics: Occurrence, effects and sources of releases to the environment in Denmark".).

Laurent, A., Bakas, I., Clavreul, J., Bernstad, A., Niero, M., Gentil, E., et al. (2014). Review of LCA studies of solid waste management systems – Part I: Lessons learned and perspectives. *Waste Management* 34(3)**,** 573-588. doi: https://doi.org/10.1016/j.wasman.2013.10.045.

Lazarevic, D., Aoustin, E., Buclet, N., and Brandt, N. (2010). Plastic waste management in the context of a European recycling society: Comparing results and uncertainties in a life cycle

perspective. *Resources, Conservation and Recycling* 55(2), 246-259. doi: https://doi.org/10.1016/j.resconrec.2010.09.014.

Lopez, G., Artetxe, M., Amutio, M., Alvarez, J., Bilbao, J., and Olazar, M. (2018). Recent advances in the gasification of waste plastics. A critical overview. *Renewable and Sustainable Energy Reviews* 82, 576-596. doi: https://doi.org/10.1016/j.rser.2017.09.032.

Meys, R., Frick, F., Westhues, S., Sternberg, A., Klankermayer, J., and Bardow, A. (2020). Towards a circular economy for plastic packaging wastes – the environmental potential of chemical recycling. *Resources, Conservation and Recycling* 162, 105010. doi: https://doi.org/10.1016/j.resconrec.2020.105010.

Miandad, R., Rehan, M., Barakat, M.A., Aburiazaiza, A.S., Khan, H., Ismail, I.M.I., et al. (2019). Catalytic Pyrolysis of Plastic Waste: Moving Toward Pyrolysis Based Biorefineries. 7(27). doi: 10.3389/fenrg.2019.00027.

NTVUganda (2013). "Eco Talk: Plastic tiles". (Uganda).

Operation Clean Sweep (2020). "PlasticsEurope Operation Clean Sweep®: Report 2019". (Brussels, Belgium).

RDC-Environment (2010). "Analyse du cycle de vie d'une bouteille PET". (Brussels, Belgium).

Rødland, E. (2019). Ecotoxic potential of road-associated microplastic particles (RAMP). *Vann* 54(3), 166-183.

Schmidt, A., Kløverpris, N.H., Bakas, I., Kjær, B.J., Vogt, R., and Giegrich, J. (2009). "Comparative life cycle assessment of two options for waste tyre treatment: material recycling versus civil engineering applications - Executive summary".).

Schwarz, A.E., Ligthart, T.N., Godoi Bizarro, D., De Wild, P., Vreugdenhil, B., and van Harmelen, T. (2021). Plastic recycling in a circular economy; determining environmental performance through an LCA matrix model approach. *Waste Management* 121, 331-342. doi: https://doi.org/10.1016/j.wasman.2020.12.020.

Shen, L., Nieuwlaar, E., Worrell, E., and Patel, M.K. (2011). Life cycle energy and GHG emissions of PET recycling: change-oriented effects. *The International Journal of Life Cycle Assessment* 16(6), 522-536. doi: 10.1007/s11367-011-0296-4.

Shen, L., Worrell, E., and Patel, M.K. (2010). Open-loop recycling: A LCA case study of PET bottle-to-fibre recycling. *Resources, Conservation and Recycling* 55(1), 34-52. doi: https://doi.org/10.1016/j.resconrec.2010.06.014.

Shonfield, P. (2008). "LCA of Management Options for Mixed Waste Plastics". (Banbury, UK Waste and Resource Action Programme (WRAP)).

Spath, P.L., Mann, M.K., and Kerr, D.R. (1999). "Life cycle assessment of coal-fired power production". (Colorado, USA).

Tait, P.W., Brew, J., Che, A., Costanzo, A., Danyluk, A., Davis, M., et al. (2019). The health impacts of waste incineration: a systematic review. n/a(n/a). doi: 10.1111/1753-6405.12939.

The Association of Plastics Recyclers (nd). *APR Design Guide®: Recyclable per APR Definition* [Online]. Washington DC, USA: The Association of Plastics Recyclers,. Available: https://plasticsrecycling.org/recycling-definitions [Accessed 27 November 2020].

Tsai, C.J., Chen, M.L., Chang, K.F., Chang, F.K., and Mao, I.F. (2009). The pollution characteristics of odor, volatile organochlorinated compounds and polycyclic aromatic hydrocarbons emitted from plastic waste recycling plants. *Chemosphere* 74(8), 1104-1110. doi: 10.1016/j.chemosphere.2008.10.041.

U.S. Department of Energy (2011). "Technology Readiness Assessment Guide". (Washington, D.C.).

United States Environmental Protection Agency (nd). "Definitions: Utilized in the Re-TRAC Connect™ State Measurement Template".).

Unwin, J., Coldwell, M.R., Keen, C., and McAlinden, J.J. (2013). Airborne Emissions of Carcinogens and Respiratory Sensitizers during Thermal Processing of Plastics. *The Annals of Occupational Hygiene* 57(3)**,** 399-406. doi: 10.1093/annhyg/mes078.

Vogelsang, C., Lusher, A.L., Dadkhah, M.E., Sundvor, I., Umar, M., Ranneklev, S.B., et al. (2020). "Microplastics in road dust – characteristics, pathways and measures". (Oslo, Norway).

Williams, P.T., and Williams, E.A. (1999). Interaction of Plastics in Mixed-Plastics Pyrolysis. *Energy & Fuels* 13(1)**,** 188-196. doi: 10.1021/ef980163x.

Wolfesberger, U., Aigner, I., and Hofbauer, H. (2009). Tar content and composition in producer gas of fluidized bed gasification of wood—Influence of temperature and pressure. *Environmental Progress & Sustainable Energy* 28(3)**,** 372-379. doi: https://doi.org/10.1002/ep.10387.

Wu, J.S. (2018). *Capital Cost Comparison of Waste-to-Energy (WTE), Facilities in China and the U.S.* Master of Science in Earth and Environmental Engineering, Columbia University.

Yamashita, K., Yamamoto, N., Mizukoshi, A., Noguchi, M., Ni, Y., and Yanagisawa, Y. (2009). Compositions of volatile organic compounds emitted from melted virgin and waste plastic pellets. *Journal of the Air and Waste Management Association* 59(3)**,** 273-278. doi: 10.3155/1047-3289.59.3.273.

Zeng, X., Ueki, Y., Yoshiie, R., Naruse, I., Wang, F., Han, Z., et al. (2020). Recent progress in tar removal by char and the applications: A comprehensive analysis. *Carbon Resources Conversion* 3**,** 1-18. doi: https://doi.org/10.1016/j.crcon.2019.12.001.

Zheng, J., and Suh, S. (2019). Strategies to reduce the global carbon footprint of plastics. *Nature Climate Change* 9(5)**,** 374-378. doi: 10.1038/s41558-019-0459-z.
